# Triglyceride-Mimetic Prodrugs of Buprenorphine Enhance Oral Bioavailability via Promotion of Lymphatic Transport

**DOI:** 10.3389/fphar.2022.879660

**Published:** 2022-04-12

**Authors:** Tim Quach, Luojuan Hu, Sifei Han, Shea F. Lim, Danielle Senyschyn, Preeti Yadav, Natalie L. Trevaskis, Jamie S. Simpson, Christopher J. H. Porter

**Affiliations:** ^1^ Medicinal Chemistry, Monash Institute of Pharmaceutical Sciences, Monash University, Parkville, VIC, Australia; ^2^ Drug Delivery, Disposition and Dynamics, Monash Institute of Pharmaceutical Sciences, Monash University, Parkville, VIC, Australia

**Keywords:** lymphatic transport, prodrug, triglyceride mimetic, buprenorphine, first-pass metabolism, oral bioavailability, opioid analgesics

## Abstract

Buprenorphine (BUP) is a potent opioid analgesic that is widely used for severe pain management and opioid replacement therapy. The oral bioavailability of BUP, however, is significantly limited by first-pass metabolism. Previous studies have shown that triglyceride (TG) mimetic prodrugs of the steroid hormone testosterone circumvent first-pass metabolism by directing drug transport through the intestinal lymphatics, bypassing the liver. The current study expanded this prodrug strategy to BUP. Here different self-immolative (SI) linkers were evaluated to conjugate BUP to the 2 position of the TG backbone via the phenol group on BUP. The SI linkers were designed to promote drug release in plasma. Lipolysis of the prodrug in the intestinal tract was examined via incubation with simulated intestinal fluid (SIF), and potential for parent drug liberation in the systemic circulation was evaluated via incubation in rat plasma. Lymphatic transport and bioavailability studies were subsequently conducted in mesenteric lymph duct or carotid artery-cannulated rats, respectively. TG prodrug derivatives were efficiently transported into the lymphatics (up to 45% of the dose in anaesthetised rats, vs. less than 0.1% for BUP). Incorporation of the SI linkers facilitated BUP release from the prodrugs in the plasma and in concert with high lymphatic transport led to a marked enhancement in oral bioavailability (up to 22-fold) compared to BUP alone. These data suggest the potential to develop an orally bioavailable BUP product which may have advantages with respect to patient preference when compared to current sublingual, transdermal patch or parenteral formulations.

## Introduction

**FIGURE 1:**
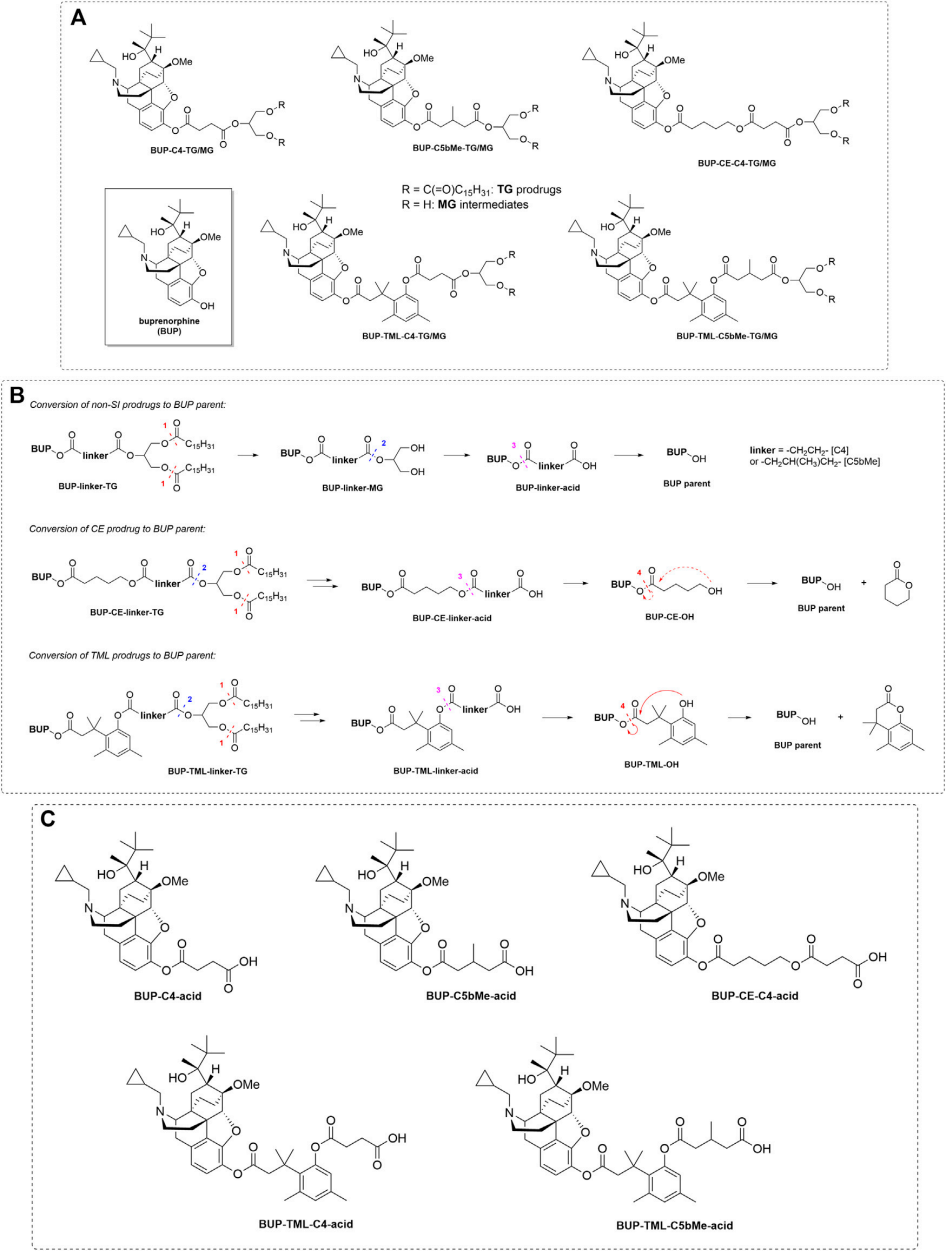
**Panel (A)** Structure of BUP, TG prodrugs and MG forms of the prodrugs (i.e., the lipolysis products after the removal of the fatty acids in the sn-1 and sn-3 positions from the prodrugs). **Panel (B)** General schematic to show the proposed mechanisms of release of drug from prodrug in plasma for both SI and non-SI containing prodrugs. The dashed lines and arrows indicate the expected mechanisms for BUP release from the MG or acid forms of the prodrugs and the numbers indicate the expected order of transition. **Panel (C)** Structures of the acid forms of the prodrugs (i.e., the intermediate products after cleavage from the MG in plasma and before breakdown of the acid form to generate BUP).

Buprenorphine (BUP, Figure 1) is a potent µ-opioid receptor partial agonist that is widely used for pain management and opioid replacement therapy (Johnson et al., 2005; Coe et al., 2019; Hale et al., 2021). Owing to high affinity for the µ-opioid receptor and slow receptor dissociation, BUP has a long duration of action, and may be used as a replacement for a number of widely used opioid analgesics, including fentanyl, oxycodone and morphine (Mercadante et al., 2009; Daitch et al., 2014; Prommer 2015). One of the main advantages of BUP is lower respiratory depression compared to fentanyl and morphine (Kress 2009). BUP has also been suggested to have a better gastrointestinal safety profile, e.g., less nausea compared to fentanyl and morphine (Wolff et al., 2012), and potentially less constipation compared to morphine (Likar et al., 2006; Prommer 2015).

**FIGURE 2:**
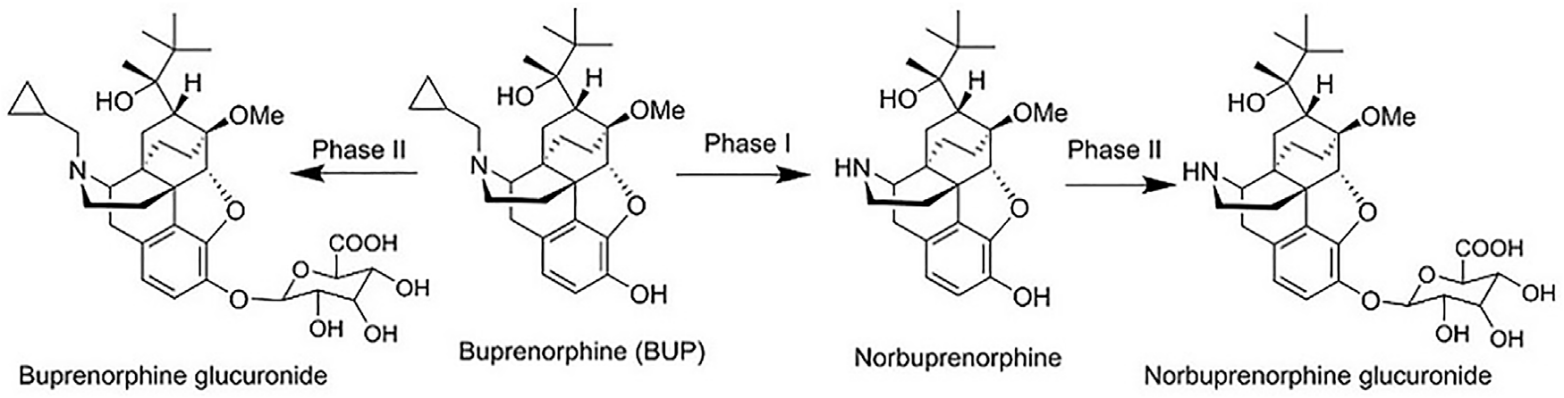
The main metabolism pathway and metabolites of BUP, including norbuprenorphine, norbuprenorphine glucuronide, and buprenorphine glucuronide.

In common with opioid analgesics including fentanyl, first-pass metabolism of BUP is extensive, limiting oral bioavailability and dictating the need for alternate delivery approaches. BUP is completely absorbed across the gut wall, but the oral bioavailability (BA) is limited (to less than 10%) by intestinal and hepatic first-pass metabolism, predominantly via two pathways in humans (Taylor et al., 2013) (Figure 2): 1) phase I metabolism via *N*-dealkylation of BUP (primarily by CYP3A4) to generate norbuprenorphine, followed by phase II metabolism (glucuronidation) of the phenol group in norbuprenorphine to generate norbuprenorphine glucuronide and 2) direct Phase II conjugation of the phenol group in BUP to generate buprenorphine glucuronide. The low oral bioavailability of BUP increases the risk of highly variable pharmacokinetics since drugs with low bioavailability are often subject to high inter-individual differences, drug-drug interactions (Fihlman et al., 2018), and food effects (Maharao et al., 2017)), and may also increase safety risks, since norbuprenorphine shows 10 fold higher respiratory depression than BUP (Strang et al., 2018).

Due to low oral bioavailability, BUP is marketed primarily as mucosal (sublingual/buccal), transdermal (patch), or injectable (e.g., intravenous/intramuscular/implant) formulations. Compared to injectables, non-injectable formulations offer better convenience for clinical use, however mucosal formulations have been documented to cause dental health concerns, potentially due to pH change in the oral cavity (Suzuki et al., 2013; Grisham et al., 2019) and transdermal patches cause skin irritation in 25–45% of patients (Prommer 2015; Serpell et al., 2016). Sublingual BUP is also commonly used as a substitute for stronger opioids such as heroin, followed by a tapering strategy to allow withdrawal. Sublingual doses, however, present a practical problem for this approach since patients can hold the dose in their mouth, leave the administering pharmacy and then remove and divert to illicit supply. The ability to develop an oral (swallowable) BUP product with high bioavailability would therefore address a range of important unmet clinical needs. In an attempt to enhance BUP oral bioavailability, previous studies have administered BUP with dietary components (e.g., ginger extracts (Joshi et al., 2017; Maharao et al., 2017)) and generally regarded as safe (GRAS) compounds (e.g., silybin (Joshi et al., 2017; Maharao et al., 2017)) in order to inhibit first-pass phase I and phase II metabolism. Some studies have shown bioavailability enhancement (Joshi et al., 2017), however these effects were moderate (absolute oral bioavailability <10%) and likely to be variable, therefore alternative approaches are required.

Re-directing drug transport from the intestine through the intestinal lymphatic system, rather than the portal blood, provides a means to improve the oral bioavailability of drugs that have high hepatic first-pass metabolism (Trevaskis et al., 2008; Yanez et al., 2011; Bala et al., 2016; Hu et al., 2016; Franco et al., 2020). In contrast to the mesenteric venous network that collects the blood from the intestine into the portal vein before passing through the liver, the intestinal lymphatics drain into the thoracic duct and empty directly into the systemic blood via the major veins in the neck. Drug transport via the intestinal lymphatics can be promoted by drug association with intestinal lipoproteins, particularly chylomicrons (CM), during transport across the enterocyte. This is because intestinal lipoproteins are relatively large (up to 1 µm in diameter) and as such do not readily pass across the continuous blood vascular endothelium, but instead preferentially traverse the more permeable lymphatic endothelial barrier (Zhang et al., 2018; Cifarelli and Eichmann 2019). Drug association with intestinal lipoproteins, and thus lymphatic transport, may occur spontaneously for highly lipophilic drug molecules (typically those with log Ds > 5 and TG solubilities >50 mg/g (Charman and Stella 1986; Porter and Charman 2001; Trevaskis et al., 2008)). For most drugs that do not exhibit such high lipophilicity, however, lipoprotein association and intestinal lymphatic transport is usually low.

To enhance lipoprotein association for less lipophilic drugs, lipophilic prodrugs can be employed. A clinically successful example of this approach is testosterone undecanoate, an aliphatic ester prodrug of testosterone (TST), a compound with very high first pass metabolism. The conjugation of TST to a C_11_ straight chain fatty acid (FA) to form testosterone undecanoate greatly enhances the lipophilicity of the parent compound thereby enhancing lipoprotein association, lymphatic transport and oral bioavailability (Tauber et al., 1986a; Tauber et al., 1986b). This product, however, results in only ∼5% oral bioavailability for TST, potentially reflecting both inefficient association of the aliphatic ester prodrug with CM, low lymphatic transport and potentially incomplete release of TST from the prodrug in the systemic circulation (Tauber, Schroder, Dusterberg and Matthes 1986b; Shackleford et al., 2003).

Drug-TG conjugates (rather than drug-FA conjugates) offer an alternate platform to promote intestinal lymphatic transport (Porter and Charman 2001; Irby et al., 2017; Markovic et al., 2019; Elz et al., 2022). A limited number of studies have described simple TG mimetics previously and these have shown moderate benefit (Amory et al., 2003; Wang et al., 2021). One of the limitations of this approach, is the need for differential stability in the gut and the systemic blood (Scriba 1995; Amory et al., 2003; Hu et al., 2016). Thus, the prodrug must integrate into typical digestive processes in the intestine, being transformed from a drug-TG into a drug-monoglyceride (MG) and this drug-MG intermediate must be stable enough to allow absorption and integration into TG re-esterification processes in the enterocyte. This in turn promotes incorporation of the re-esterified prodrug into CM assembly processes alongside endogenous TG. After transport through the lymph into the systemic blood, this process must then be reversed and drug released from the re-esterified material in order to exert pharmacologic activity.

Previous studies in our laboratory have shown for TST that this can be achieved using appropriately designed self-immolative (SI) linkers to fine tune the stability profiles both pre- and post- absorption (Hu et al., 2016). This resulted in increases in oral bioavailability for TST that were an order of magnitude higher than that achieved with TST undecanoate. In the current study, therefore, we applied similar design concepts to BUP, realising that the conjugation point in BUP (phenol) is different to the group employed previously for TST and the molecular weight of BUP is higher than that of the molecules employed previously, raising questions as to whether the additional molecular weight imparted by the pro-moiety may limit intestinal permeability and absorption. As shown in Figure 1A, here the BUP phenol was conjugated to a TG backbone via a range of different linkers. BUP-C4-TG contained a four-carbon straight chain spacer; BUP-C5bMe-TG was designed to improve intestinal stability due to the steric effects of the beta substituted methyl group and BUP-CE-C4-TG, BUP-TML-C4-TG and BUP-TML-C5bMe-TG were designed to enhance plasma release via the incorporation of a SI group. In this case both SI groups were expected to self-immolate via cyclisation (Figure 1B). The CE (cyclising ester) SI group is a relatively simple aliphatic chain designed to cyclise to form a 6 membered ring. The TML (tri-methyl lock) group is designed to also promote cyclisation and for the methyl substituents to promote that process thereby potentially increasing the release of BUP after entry into the systemic circulation. The prodrugs were first evaluated *in vitro* via incubation with simulated intestinal fluid (SIF) to map lipolysis from the TG derivative to the corresponding MG derivative (Figure 1A) and to subsequently examine the stability of the MG derivative in the intestine. Studies were also conducted with the intact TG prodrug in the presence of plasma to simulate conversion of the re-esterified prodrug to parent drug or to the intermediate “acid” form (where the drug remains attached to the SI group and linker) in the blood (Figures 1B,C). Animal studies were then conducted to examine the extent of lymphatic transport and oral bioavailability of BUP after administration of the prodrug. The work aims to 1) define whether the general trends seen previously with TG-mimetic prodrugs of TST are also seen in a molecule where conjugation is via a phenol, 2) examine the impact of the increase in molecular weight of BUP on the absorption and lymphatic transport of the TG-mimetic prodrugs and 3) evaluate the utility of a cyclising ester self immolative group.

**TABLE 1:** Molecular weight (MW) and cLogP values of BUP, prodrugs, and expected MG-like intermediates generated upon hydrolysis by digestive fluid in the GI lumen. Data were calculated OSIRIS Datawarrior (Version 5.5.0).

	MW of TG prodrug	cLogP of TG prodrug	MW of MG intermediate	cLogP of MG intermediate
Parent BUP	468	4.3	NA	NA
BUP-C4-TG	1119	17.3	642	3.6
BUP-C5bMe-TG	1147	18.0	670	4.3
BUP-CE-C4-TG	1219	18.2	742	4.0
BUP-TML-C4-TG	1323	20.7	846	7.0
BUP-TML-C5bMe-TG	1351	21.4	874	7.7

## Materials and Methods

### Chemicals, Synthesis of BUP Prodrugs, and Formulation Preparation

The buprenorphine (BUP) prodrugs were synthesised from BUP (Tasmanian Alkaloids, Tasmania, Australia) as described in the Supplementary Material. Oleic acid, Tween 80, lipoprotein lipase, and porcine pancreatin were purchased from Sigma-Aldrich, MO, United States. Acetonitrile for liquid chromatography was purchased from Merck Pty Limited, VIC, Australia. Ultrapure water was obtained from a Milli-Q™ system (Millipore, MA, United States). All other chemicals were analytical grade or above. Lipid-based formulations of the prodrugs or BUP for *in vivo* studies were assembled as described previously (Han et al., 2014) using ultrasonication. Formulations for each animal in the lymphatic transport studies contained 50 µg of prodrug or parent BUP, 40 mg oleic acid, 25 mg Tween 80 and 5.6 ml phosphate buffered saline (PBS, pH 7.4). Formulations for each animal in the oral bioavailability studies contained 50 µg of prodrug or 20 µg of parent BUP, 40 mg oleic acid, 25 mg Tween 80 and 2 ml PBS. The IV formulation was a solution of 20 μg/ml BUP in PBS (10 µg in 0.5 ml for each animal).

### *In Vitro* Digestion of Prodrugs by Porcine Pancreatin

To study the hydrolysis of TG prodrugs in the intestinal lumen, *in vitro* digestion of BUP prodrugs was performed via incubation with pancreatic lipase (1,000 IU/ml) as described previously (Hu et al., 2016). Briefly, the lipase solution was prepared by dispersion of porcine pancreatin (10,000 IU/ml) in a lipolysis buffer (pH 6.5) containing tris-maleate (2 mM), CaCl_2_ (1.4 mM) and NaCl (150 mM), followed by centrifugation at 2,000 *g* for 15 min at 5°C to provide the lipase solution as the supernatant. To start the hydrolysis, 100 µl of the lipase solution was added to a mixture of 20 µl of prodrug solution (0.1 mg/ml dissolved in acetonitrile) and 900 µl of simulated intestinal micellar solution (3 mM of sodium taurodeoxycholate and 0.75 mM of phosphatidylcholine dispersed in lipolysis buffer). The solution was incubated at 37°C. Samples (20 µl) of the incubation solution were taken at 0, 5, 10, 15, 30, 60, 90, 120 and 180 min post incubation and added to 180 µl of acetonitrile to stop lipolysis. The mixture was vortexed and centrifuged at 4,500 g for 5 min to precipitate proteins prior to analysis. The supernatant was analysed by HPLC-MS/MS for the potential products (Figure 1) of prodrug hydrolysis as described in the Supplementary Material. These were the MG form (where the fatty acids are hydrolysed in the *sn*-1 and *sn*-3 position of the prodrug), the “acid” form (where the drug-linker-COOH is cleaved from the glyceride backbone) and BUP.

### *In Vitro* Incubation of Prodrugs With LPL Supplemented Plasma

To probe the release of BUP from the TG prodrugs in the systemic circulation, BUP prodrugs were incubated with rat plasma supplemented with lipoprotein lipase (LPL, 200 IU/ml) as previously described (Hu et al., 2016). LPL is a key enzyme required for the hydrolysis of lipoprotein associated TG in the systemic circulation and is therefore expected to be a key contributor to lipolysis of the re-esterified BUP TG construct in plasma, largely via liberation of FAs in the *sn*-1 and the *sn*-3 position of the TG-mimetic prodrug, prior to drug release from the *sn*-2 position via esterase hydrolysis. LPL is active in plasma but is tethered to the luminal surface of vascular endothelial cells under physiological conditions (Bensadoun 1991). In the current *in vitro* studies, rat plasma was therefore supplemented with LPL to better reflect the *in vivo* situation. To start hydrolysis, 10 µl of LPL solution (10,000 IU/ml) was added to a mixture of 10 µl of prodrug solution (0.1 mg/ml dissolved in acetonitrile) and 500 µl of blank Sprague Dawley rat plasma. The solution was incubated at 37°C. Samples (20 µl) of the incubation solution were taken at 0, 5, 15, 30, 60, 90, 120 and 180 min post incubation and added to 180 µl of ACN to stop lipolysis. The mixture was vortexed and centrifuged at 4,500 g for 5 min to precipitate proteins prior to analysis. The supernatant was analysed by HPLC-MS/MS for the potential products (e.g., MG form, acid form, and BUP) of prodrug hydrolysis (see Supplementary Material).

### Animal Care During *In Vivo* Experiments

All animal experiments were approved by the Monash Institute of Pharmaceutical Sciences animal ethics committee and were conducted in accordance with the Australian and New Zealand Council for the Care of Animals in Research and Teaching guidelines. Male Sprague-Dawley rats (270–320 g) were maintained on a standard diet and fasted overnight with free access to water prior to experiments. In experiments where surgical anaesthesia was required (i.e., for the cannulation of the carotid artery, jugular vein, and mesenteric lymph duct), rats were anaesthetised using a combination of ketamine, xylazine and acepromazine and placed on a heated pad at 37°C as described previously (Johnson et al., 2003). At the end of experiments rats were euthanised via intraperitoneal, intra-arterial or intravenous (when an indwelling cannula was present) administration of 100 mg pentobarbitone.

### Lymphatic Transport Studies

Lymphatic transport studies were conducted as previously described (Han et al., 2014) using a rat model with cannulas inserted into the duodenum (for formulation administration and rehydration) and mesenteric lymph duct (for lymph collection) (Trevaskis et al., 2015). Post-surgery, rats were re-hydrated for 0.5 h via intraduodenal infusion of normal saline at 2.8 ml/h and the animals remained anaesthetized throughout the experimental period. The lipid formulations were then infused into the duodenum at a rate of 2.8 ml/h for 2 h, after which the infusion was changed back to 2.8 ml/h normal saline for the remainder of the experiment. Lymph was continuously collected for up to 6 h following initiation of formulation administration into pre-weighed Eppendorf tubes containing 10 µl of 1,000 IU/ml heparin, and the collection tubes were changed hourly. Aliquots (20 µl) of each hourly collected lymph sample were immediately transferred into new Eppendorf tubes and stored at –20°C prior to analysis. The mass transport of BUP or prodrugs into lymph during each 1 hour collection period was calculated from the product of the volume of lymph collected and the measured concentration in lymph, respectively. The cumulative percentage of lymphatic transport over time was calculated as the mole ratio of all BUP-related species in lymph relative to the equivalent moles of drug or prodrug administered, where BUP or total BUP-related species in lymph following administration of TG mimetic prodrugs were quantified as described under “Data analysis” and in the Supplementary Material.

### Bioavailability Studies

The systemic exposure of BUP was examined after oral administration of BUP or BUP prodrugs in carotid artery cannulated conscious rats (for the group of animals that received BUP intravenously (IV), the jugular vein was also cannulated for IV infusion). After surgery and recovery, the rats were housed in Culex Automated Blood Sampler systems (BASi, West Lafayette, Indian, United States) with free movement as described previously (Mannila et al., 2012). After drug administration via bolus oral gavage or IV infusion (over 5 min), blood samples (150 µl) were taken from the carotid artery cannula 5 min prior to administration and up to 24 h post-dosing and centrifuged at 4,500 g for 5 min to separate plasma. During the blood sample collection period the rats had free access to water at all times but remained fasted for a further 8 h following drug administration. Plasma samples were stored at −20°C prior to analysis for BUP as described in the Supplementary Material. The area under the plasma concentration-time curves from zero to designated time intervals (AUC_0-t_) were calculated using the linear trapezoidal method. The oral bioavailability of BUP was estimated via comparison of dose normalised AUC_0–6h_ following oral dosing of BUP or TG prodrugs with the AUC_0-inf_ following IV infusion of BUP (AUC_0-inf_ was calculated via the addition of C_t_/k to AUC_0-t_, where C_t_ is the last observed quantifiable concentration and k is the terminal phase elimination rate constant). Truncated AUCs (AUC_0–6h_) were used for the oral pharmacokinetic studies due to the presence of a second peak in the BUP plasma concentration versus time profile, most likely reflecting enterohepatic recycling (Ohtani et al., 1994).

### Data Analysis

**FIGURE 3:**
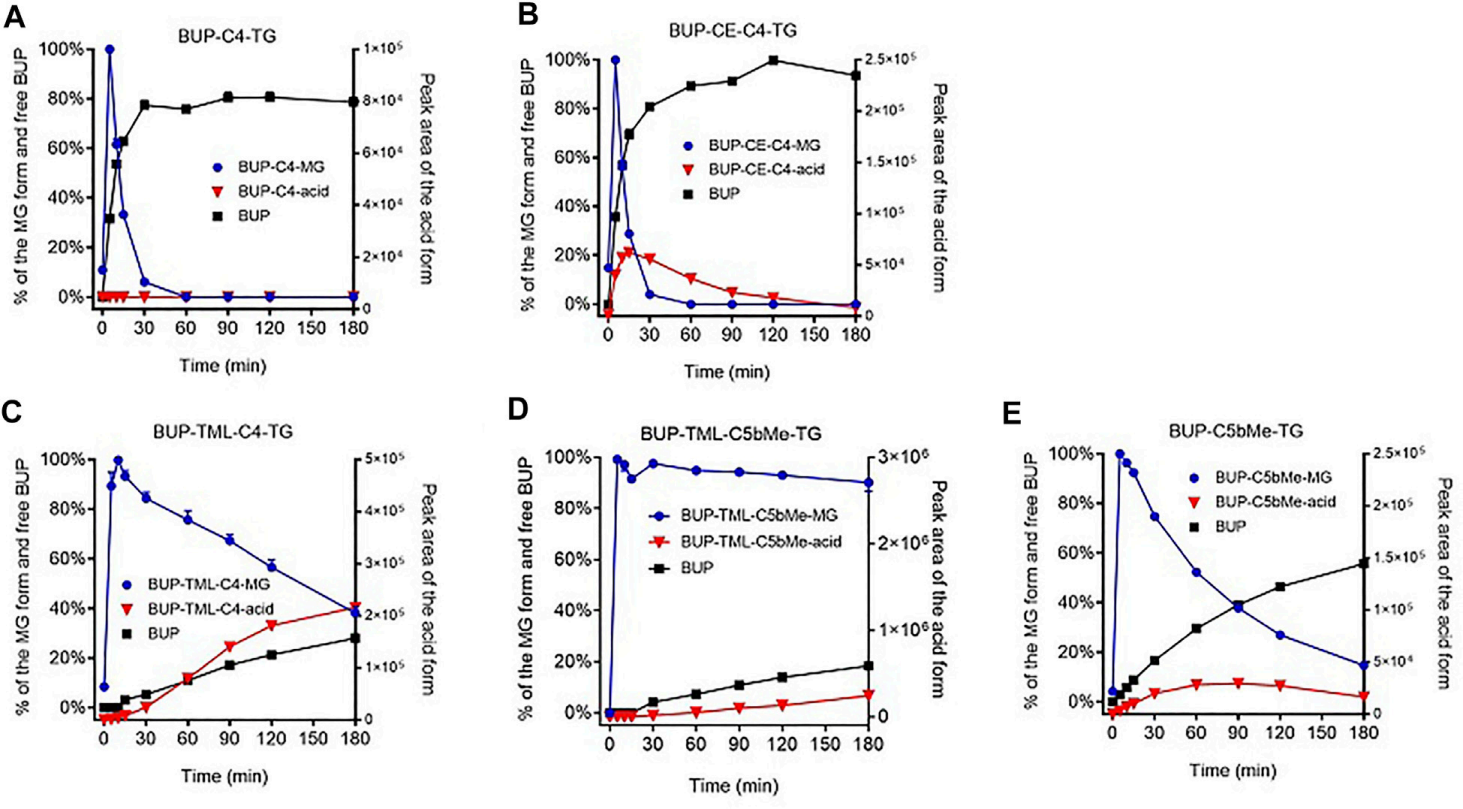
GI stability profiles of BUP prodrugs. Data are shown as % mass change (Mean ± SEM, *n* = 3) for MG-like forms and parent BUP, or as arbitrary peak area for BUP-linker acid forms, upon *in vitro* incubation of BUP-C4-TG **(A)**, BUP-CE-C4-TG-TG **(B)**, BUP-TML-C4-TG **(C)**, BUP-TML-C5bMe-TG **(D)**, and BUP-C5bMe-TG **(E)**, with simulated intestinal fluid (SIF) containing porcine pancreatin.

Hydrolysis of prodrugs in SIF and plasma: Percent BUP generation was calculated as the ratio of the molar concentration of BUP produced during incubation relative to the molar concentration of prodrug at the initiation of the incubation. Absolute quantification of the hydrolysis products, i.e., MG derivatives and BUP-linker-COOH forms (“acid” forms) was not attempted due to lack of authentic standards. Instead, the profiles for these species were plotted based on the peak areas obtained from HPLC-MS. To illustrate changes to the relative concentration of MG derivatives, the highest peak area (typically at the first sampling time point, i.e., 5 min) was nominally set as 100% (Figures 3, 5) on the basis that previous studies suggest that lipolysis is rapid and complete. To illustrate changes to the BUP-linker-acid forms, the relative changes were plotted as the peak areas on the right-hand *Y* axis in Figures 3 and 5.

Statistical methods: Statistical differences were determined by ANOVA followed by Dunnett’s test for multiple comparisons at a significance level of *p* = 0.05, using GraphPad Prism for Windows V9.0.1 (GraphPad Software Inc., CA, United States).

## Results

### Hydrolysis of BUP Prodrugs in SIF

As shown in Figure 3, the hydrolysis of TG prodrugs in SIF (containing porcine pancreatin) rapidly produced species with molecular weights consistent with that of the corresponding MG forms of the prodrugs (i.e., BUP-C4-MG, BUP-CE-C4-MG, BUP-TML-C4-MG, BUP-TML-C5bMe-MG, and BUP-C5bMe-MG, Figure 1). Subsequently, the stability of the MG intermediates differed significantly between groups. The concentrations of BUP-C4-MG and BUP-CE-C4-MG decreased rapidly over time such that only trace quantities were detected after 30 min incubation (Figures 3A,B). This was accompanied by increases in the concentration of released BUP to more than 80% over 3 h, with a transient presence of BUP-CE-C4-acid form. In contrast, the concentrations of the MG intermediates for BUP-TML-C4-TG, BUP-TML-C5b-TG and BUP-C5bMe-TG decreased more slowly over time (Figures 3C–E). BUP-TML-C5bMe-MG was particularly stable and the majority (>80%) remained at the end of the 3 h incubation period (Figure 3D). Degradation of the MG intermediates of the latter three prodrugs was also accompanied by increases in BUP-linker-acid forms and released BUP, but the production of BUP (quantified against authentic standards) was lower compared to BUP-C4-TG and BUP-CE-C4-TG.

### Lymphatic Transport and Bioavailability

**FIGURE 4:**
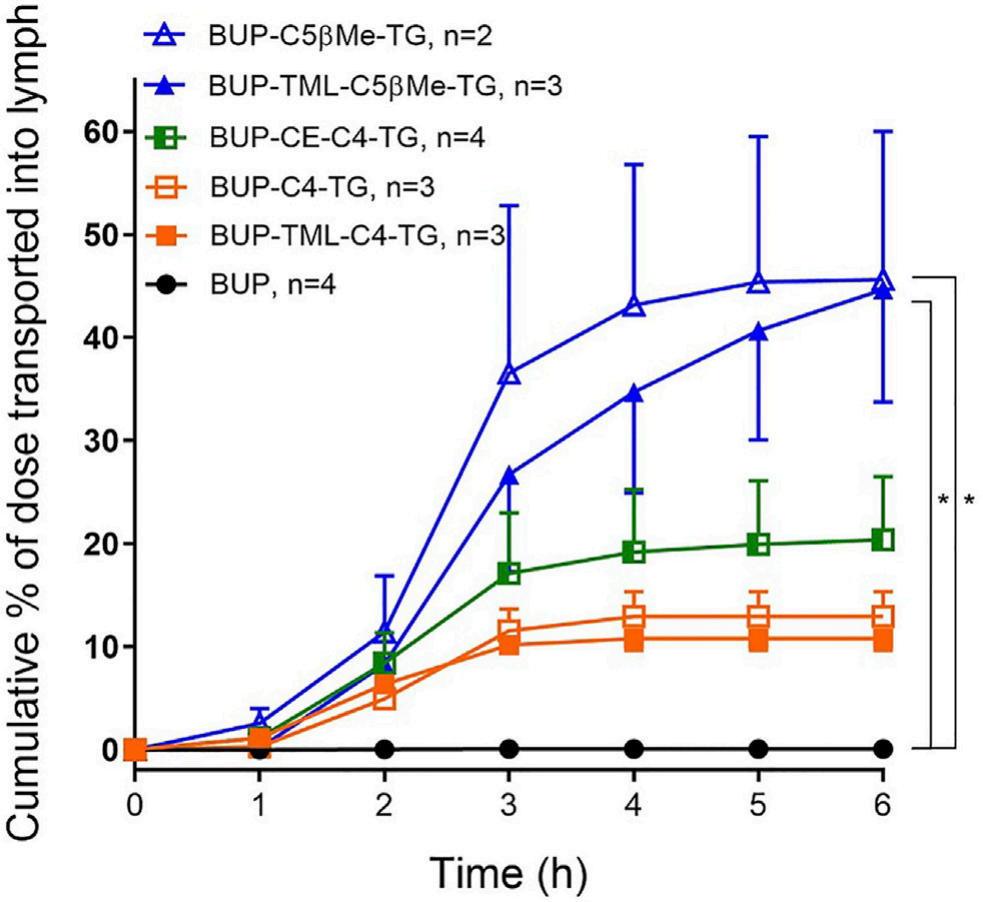
Cumulative lymphatic transport of total BUP related derivatives (% of administered dose) versus time in anaesthetised, mesenteric lymph duct cannulated rats following intraduodenal infusion of formulations from 0 to 2 h. Formulations contained 50 µg of BUP or prodrugs, dispersed in 40 mg oleic acid, 25 mg Tween 80 and 5.6 ml PBS. Data are presented as Mean ± SEM when *n* = 3 or 4 (as labelled for individual groups) or as Mean ± Range in the case of BUP-C5bMe-TG when *n* = 2. * indicates statistically significantly higher lymphatic transport of BUP-C5bMe-TG and BUP-TML-C5bMe-TG compared to parent BUP (*p* < 0.01).

The lymphatic transport of BUP after administration of the parent drug was extremely low, and only 0.058% of the administered dose (Figure 4). In contrast, following administration of all the prodrugs, the lymphatic transport of BUP related derivatives was increased (>10% of the administered dose, Figure 4). The cumulative % dose of BUP transported into the mesenteric lymph over 6 h was 12.9% for BUP-C4-TG, 20.4% for BUP-CE-C4-TG, 10.8% for BUP-TML-C4-TG, 44.7% for BUP-TML-C5bMe-TG and 45.6% for BUP-C5bMe-TG. The latter two prodrugs demonstrated statistically significant differences (*p* < 0.01) when compared to BUP group).

### Liberation of BUP from Prodrugs in Plasma

**FIGURE 5:**
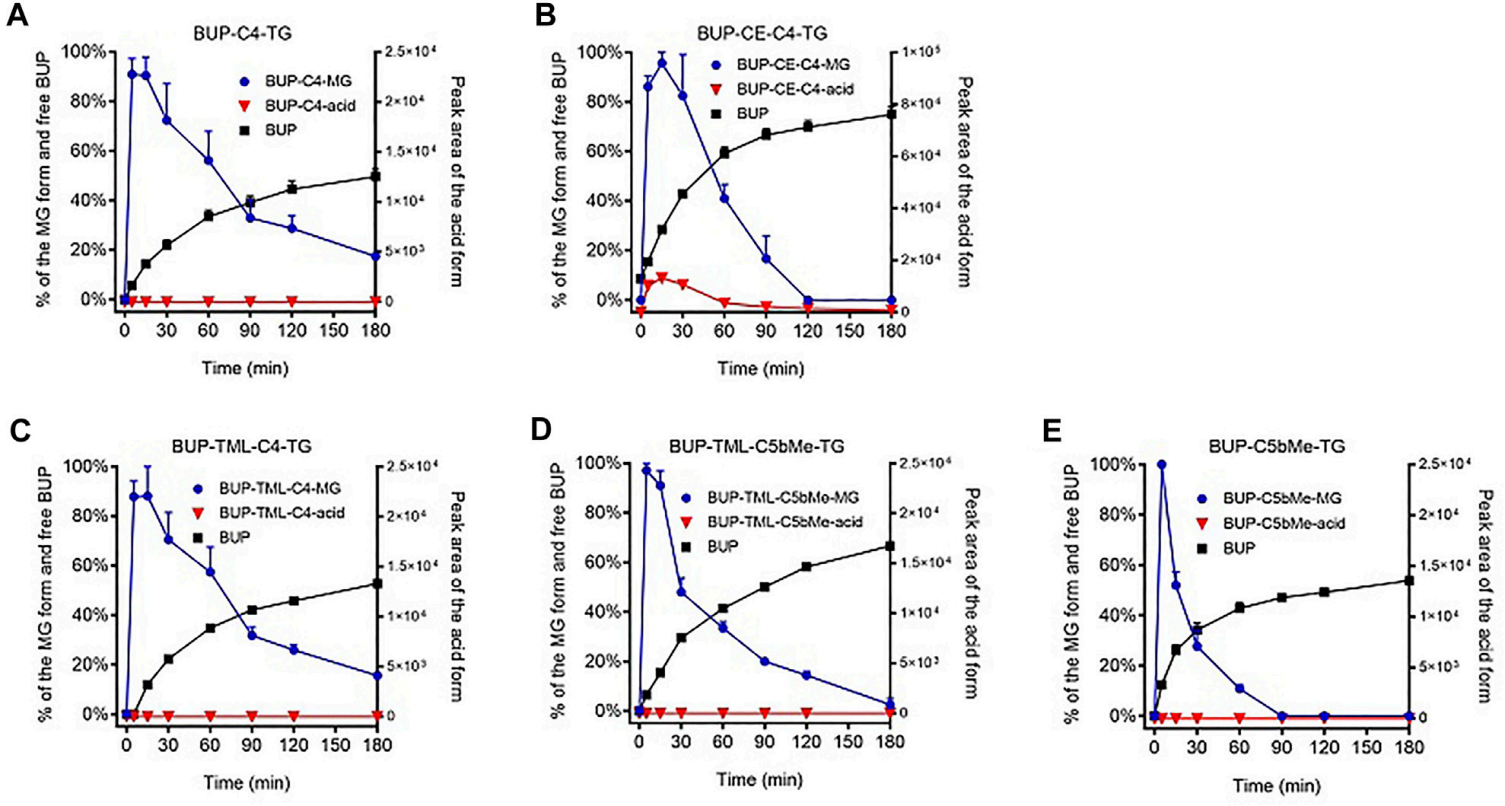
*In vitro* prodrug conversion profile. Data (Mean ± SEM, *n* = 3) are shown as % mass change of MG-like forms and parent BUP, or as peak area for BUP-linker-acid forms, upon *in vitro* incubation of BUP-C4-TG **(A)**, BUP-CE-C4-TG-TG **(B)**, BUP-TML-C4-TG **(C)**, BUP-TML-C5bMe-TG **(D)**, and BUP-C5bMe-TG **(E)** with rat plasma supplemented with lipoprotein lipase (LPL).

To evaluate the potential for the re-esterified prodrugs to liberate BUP in the systemic circulation after transport through the lymphatics, the prodrugs were incubated with plasma *in vitro*. Similar to the hydrolysis profiles in pancreatin containing SIF, incubation of TG prodrugs with LPL supplemented rat plasma rapidly produced species with molecular weights consistent with that of the corresponding MG forms of the prodrugs. Subsequently, the concentration changes of the MG forms in plasma, however, differed from the profiles in the SIF incubation. In the plasma, BUP-C4-MG (Figure 5A), BUP-TML-C4-MG (Figure 5C), BUP-TML-C5bMe-MG (Figure 5D) and BUP-C5bMe-MG (Figure 5E) decreased over time and this coincided with increases in the concentration of released BUP (>50% over 3 h). However, no BUP-linker-acid forms were detected. Only in the case of BUP-CE-C4-MG, was the presence of BUP-CE-C4-acid transiently detected (Figure 5B). The rates of MG degradation in plasma (apparent half-lives between 15 and 60 min) appeared to be less differentiated across the prodrugs, compared to their degradation profiles in SIF hydrolysis (where apparent half-lives ranged from <10 min to >>3 h). Pleasingly, BUP release from the two TML-linker containing prodrugs, i.e., TML-C4 and TML-C5bMe, was much more facile in plasma (with 53 and 67% BUP released over 3 h, Figures 5C,D) compared to hydrolysis in SIF over the same period (28 and 18%, respectively, Figures 3C,D, respectively). The data suggest the potential to strike the correct balance between intestinal stability and plasma instability/release.

### Bioavailability Studies

**FIGURE 6:**
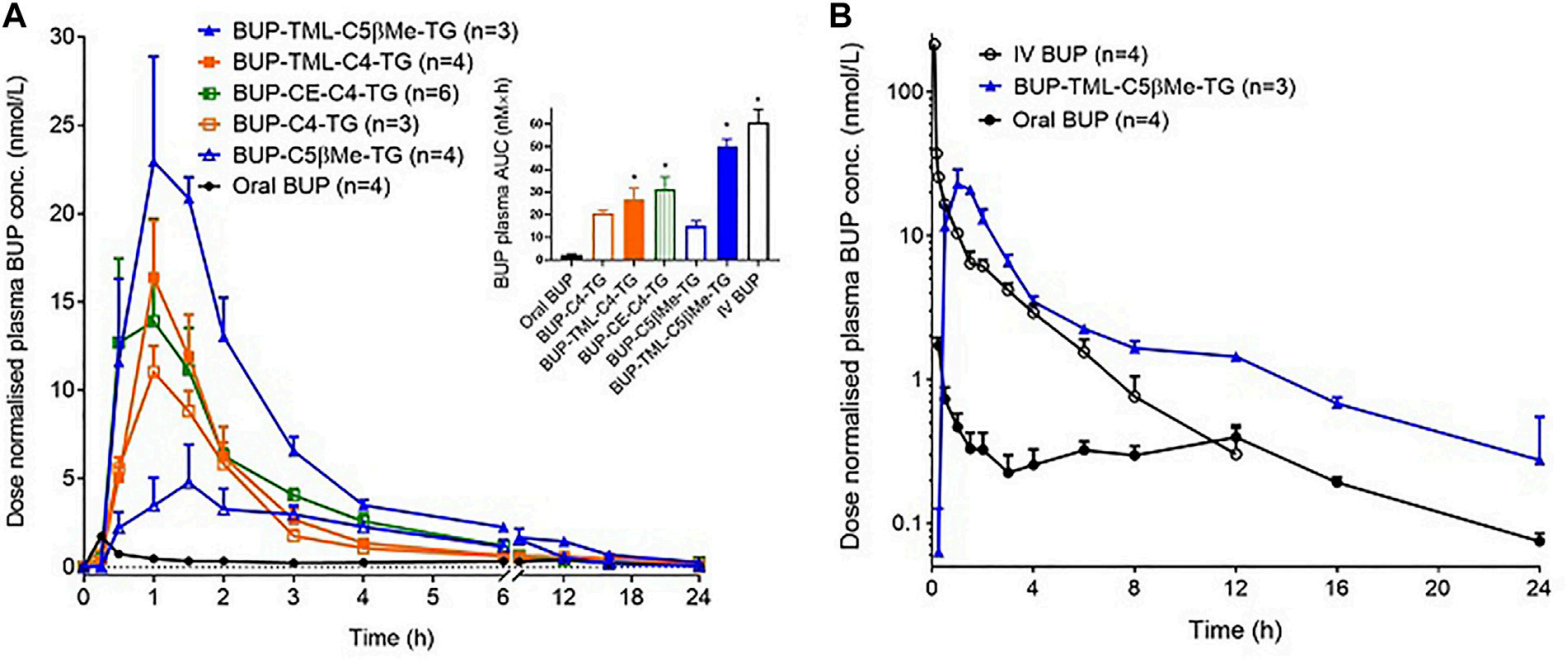
Dose-normalised BUP plasma concentrations following oral gavage **(A)** or intravenous infusion over 5 min **(B)** of formulations to conscious, carotid artery cannulated rats (the data for oral BUP and oral BUP-TML-C5bMe-TG are replicated in Panel B for comparison to IV dosing). Oral formulations contained 20 µg of BUP or 50 µg of BUP prodrugs dispersed in 40 mg oleic acid, 25 mg Tween 80 and 2 ml PBS. The IV formulation contained 10 µg of BUP dissolved in 0.5 ml PBS. Doses are normalized to a 0.06 mg/kg equivalent dose of BUP. Data are presented as Mean ± SEM (*n* = 3–6, as labelled for individual groups). The embedded bar chart in **(A)** shows the plasma AUC values (AUC_0-inf_ for IV BUP, and AUC_0–6h_ for other groups), and * indicates the value is statistically significantly higher than oral BUP group (*p* < 0.05).

Pharmacokinetic studies were conducted in conscious rats. Figure 6 and Table 2 show the dose-normalised BUP plasma concentration profiles and pharmacokinetic parameters following oral or IV administration of BUP or prodrugs. In all four animals that received an oral bolus dose of BUP by gavage, plasma concentrations reached a peak rapidly (15 min) post-dose, however, oral bioavailability was low (3.7% compared to IV), realising that truncated AUCs were employed and so this may not accurately reflect absolute bioavailability. The three SI containing prodrugs (but not BUP-C4-TG or BUP-C5bMe-TG) statistically significantly (*p* < 0.05) enhanced the plasma AUC_0–6h_ of BUP, by 12–22 fold compared to oral administration of parent BUP. BUP-TML-C4-TG and BUP-CE-C4-TG provided for moderate increases in BUP exposure (12 and 14 fold, respectively), and the self-immolative linkers (TML-C4 and CE-C4) appeared to slightly (but statistically insignificantly) improve the plasma AUC of BUP compared to BUP-C4-TG. BUP-C5b-Me-TG produced the smallest AUC improvement (6.6 fold, not statistically significant compared to BUP alone), in spite of the fact that the lymphatic transport of BUP-C5bMe-TG was the highest among all prodrugs. In contrast, the TML self-immolative group (in BUP-TML-C5βMe-TG) dramatically enhanced systemic exposure of BUP (22 fold) resulting in similar exposure to that obtained after IV administration (83% BA) (Figure 6B).

**TABLE 2:** Summary of PK parameters following oral gavage or IV infusion of formulations to conscious, carotid artery cannulated rats. Doses are normalized to a 0.06 mg/kg equivalent dose of BUP. Data are presented as Mean ± SEM.

	IV BUP (*n* = 4)	Oral BUP (*n* = 4)	BUP-C4-TG (*n* = 3)	BUP-C5bMe-TG (*n* = 4)	BUP-CE-C4-TG (*n* = 6)	BUP-TML-C4-TG (*n* = 4)	BUP-TML-C5bMe-TG (*n* = 3)
Dose (µg)	10	20	50	50	50	50	50
AUC (nM × h) (0-inf for IV; 0–6 h for other groups)	60.7 ± 5.8^a^	2.27 ± 0.44	20.6 ± 1.3	14.9 ± 2.3	31.2 ± 5.4^a^	26.7 ± 5.0^a^	50.1 ± 3.3^a^
C_max_ (nM)	215 ± 44^a^	1.73 ± 0.23	11.4 ± 1.2	5.88 ± 1.5	17.3 ± 5.2	16.4 ± 3.2	25.5 ± 4.1
T_max_ (h)	0.08 ± 0.00	0.25 ± 0.00	1.17 ± 0.17	1.88 ± 0.75^b^	1.33 ± 0.21	1.00 ± 0.00	1.33 ± 0.17
BA^c^ (% of IV)	100%	3.7%	34%	25%	51%	44%	83%
Fold increase of oral BA	N/A	1	9.1	6.6	14	12	22

a Significantly higher AUC or C_max_ than oral BUP (*p* < 0.05).

b Significantly longer T_max_ compared to oral BUP (*p* < 0.05).

c Bioavailability (BA) was estimated as the ratio of dose-normalised plasma AUC_0–6h_ of BUP following oral dosing of BUP or BUP prodrugs versus the plasma AUC_0-inf_ obtained following IV dosing of BUP, where accurate absolute bioavailability could not be calculated due to second peaks (enterohepatic recycling) in the plasma concentration profiles in oral groups.

## 4 Discussion

First pass metabolism in the liver is a barrier to effective oral delivery for a range of drugs and drug candidates. We and others have previously shown that TG mimetic prodrugs are able to re-direct drug transport into the intestinal lymphatic system after oral administration (Garzonaburbeh et al., 1983; Sugihara et al., 1988; Amory et al., 2003; Han et al., 2014; Cao et al., 2021) and that this provides an effective means to circumvent the liver and limit first pass reductions to bioavailability (Hu et al., 2016). Previous studies have described methods to produce these prodrugs by enabling drug conjugation to a TG backbone via carboxylic acids (Garzonaburbeh et al., 1983; Sugihara et al., 1988; Han et al., 2014), alcohols (Amory et al., 2003; Hu et al., 2016) and most recently sulfonamides (Cao et al., 2021). In the current study we extend these studies to BUP, where conjugation occurs via a phenol and where the MW of the parent BUP (468 Da) is larger than previous examples, and thus where addition of a pro-moiety pushes molecular weight beyond typical rule-of-5 boundaries (∼642–874 Da). Furthermore, a new SI linker (cyclising ester) has also been explored. The data suggest that the TG-mimetic prodrug technology, in combination with appropriate self immolative (SI) linkers, can be effectively applied to BUP to provide for significant lymphatic transport and robust oral bioavailability. The main findings from the study are discussed below.

### The GI Stability of the BUP Prodrug is an Important Determinant of Lymph Transport

All five TG prodrugs of BUP underwent almost instantaneous lipolysis (within minutes) to generate corresponding BUP-MG equivalents upon contact with pancreatin-containing SIF (Figure 3). This is consistent with previous observations on the lipolysis of other TG mimetic compounds of structurally unrelated drugs such as mycophenolic acid (Han et al., 2014) and testosterone (Hu et al., 2016). The data confirms that intestinal digestive enzymes (most likely pancreatic lipase) are able to identify the TG scaffold and cleave the FA molecules at *sn*-1 and *sn*-3 position regardless of the presence of a drug molecule rather than a FA at the *sn*-2 position. This suggests that the chemical space that can be tolerated by adjacent lipolysis enzymes is potentially large and that a relatively wide range of parent drug and linker chemistries may be compatible with the TG template without impairing the capacity of the initial lipolysis step. This is important, since this step is a likely prerequisite for downstream events and previous studies have shown that inhibiting lipolysis results in poor absorption of a TG prodrug of MPA (Han et al., 2015).

**FIGURE 7:**
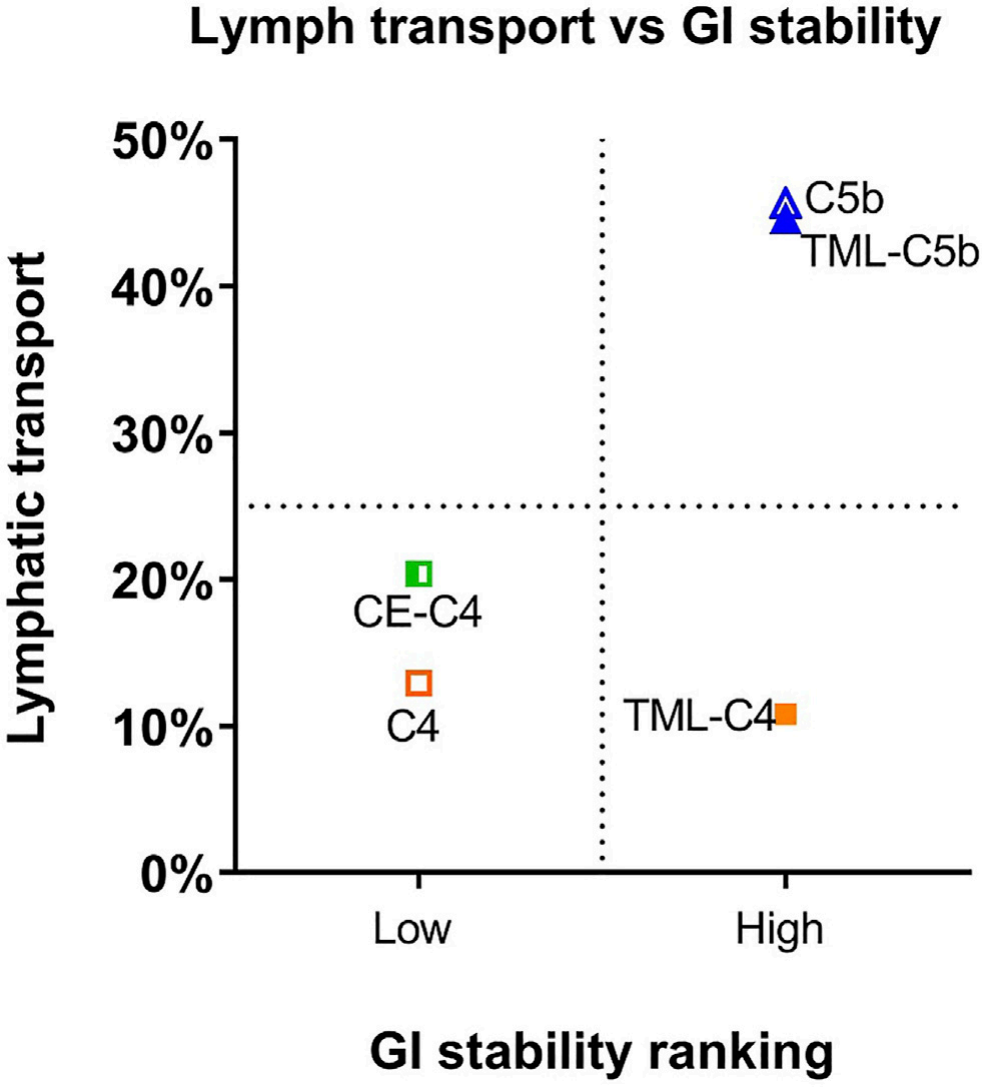
Comparison of lymphatic transport and GI lumen stability of BUP prodrugs, where GI lumen stability of the MG intermediates is categorized as low (apparent half-life < 10 min) and high (apparent half-life > 1 h). GI stability ranking, rather than exact half-lives are used for *x*-axis because the rapid degradation of BUP-C4-MG and BUP-CE-C4-MG prevents accurate calculation of half-lives.

After cleavage of the two FAs from the prodrug, the MG equivalents of the five constructs showed very different stability profiles, and this likely contributed to the differences seen in lymphatic transport of the prodrugs (Figure 7). BUP-C4-MG and BUP-CE-C4-MG degraded very rapidly releasing BUP in SIF (>50% BUP liberated within 10–15 min), suggesting that these linkers are highly labile in the presence of GI luminal digestive enzymes. The *in vitro* GI stability data is consistent with the lower lymphatic transport of these two prodrugs (12.9–20.4%), and is also consistent with previous observations for TST prodrugs where similarly unstable TST-C5-TG and TST-ASI-C5-TG prodrugs showed lower lymphatic transport than more stable prodrugs. In contrast, BUP-TML-C4-MG was more stable in simulated GI fluids (more than half of the MG intermediate remained after 2 h). This likely reflects steric hindrance of enzyme access imparted by the bulky TML structure, preventing the degradation of the MG intermediates in the GI lumen. In spite of improved GI stability, however the lymphatic transport of BUP-TML-C4-TG was only moderate (10.8%), suggesting perhaps, that the bulky TML may also impair transfer to the site of re-esterification or reduce enzyme recognition for TG re-synthesis in the enterocyte (thereby limiting CM incorporation).

In order to better stabilise the MG intermediates, a substituted C5bMe linker was employed. Previous studies with testosterone suggest that the addition of a methyl substituent on the beta carbon has the potential to stabilize the MG derivative to GI hydrolysis, but not to limit subsequent re-esterification (Hu et al., 2016). Consistent with these previous studies the C5bMe linker prolonged apparent half-life in SIF, from less than 10 min (in the case of BUP-C4-MG) to more than 1 h (in the case of BUP-C5bMe-MG). The increase in GI stability was also reflected in a significant increase in lymphatic transport of BUP-C5bMe-TG (∼45% dose). Similar to the trends observed for BUP-TML-C4-MG, addition of the TML self immolative to the C5bMe linker to form BUP-TML-C5bMe resulted in enhanced GI stability and more than 80% of BUP-TML-C5bMe-MG remained intact after 3 h of digestion in SIF. Unlike BUP-TML-C4-TG, however, the lymphatic transport of BUP-TML-C5bMe-TG was high (∼45%). One potential explanation for this apparently anomalous result is that the extra -CH (CH_3_)- in the TML-C5bMe linker may help to restore TG re-esterification capacity by changing transport or partitioning in the endoplasmic reticulum or by pushing the TML ring structure further away from the glyceride backbone, increasing access of the monoacylglycerol and diacylglycerol acyl transferases required for TG re-esterification (Shi and Cheng 2009).

### Release of BUP from the Re-Esterified Prodrug Controls Active Drug Availability in the Systemic Circulation

Release of BUP from the re-esterified BUP-TG prodrugs is required to allow BUP to exert its pharmacological activity. This is expected to occur in two steps. The first is cleavage of the two FA molecules in the *sn*-1 and *sn*-3 positions to reveal the MG equivalents, a reaction equivalent to lipolysis in the GI lumen, except that in the plasma this is mediated by lipoprotein lipase (LPL) rather than pancreatic lipase in the intestine. LPL is tethered to the vascular capillary endothelium *in vivo* and is responsible for the hydrolysis of dietary TG contained in circulating lipoproteins (Mead et al., 2002; Merkel et al., 2002). After the initial lipolysis of the TG prodrugs, the second step is parent BUP release from the MG derivative in a process likely driven by non-specific carboxylesterases (carboxylic ester hydrolases).

The first step (TG to MG hydrolysis) appears to be a prerequisite for the second step (parent drug release). This is based on previous (unpublished) observations on MPA and TST prodrugs that 1) *in vitro* incubation of TG prodrugs (with long chain FAs at the *sn*-1 and *sn*-3 position) with blank plasma does not result in generation of MG intermediates or liberation of parent drugs; and 2) *in vivo* studies reveal that inhibition of LPL (by IV infusion of the lipase inhibitor orlistat) blocks the release of drug from TG prodrugs after IV administration. In contrast, enhanced release of tethered LPL into the circulation by IV infusion of heparin accelerates TG prodrug hydrolysis and release of parent drug. That said, whilst hydrolysis of the TG prodrug to the MG derivative is critical, it does not appear to be a significant limitation as it occurs rapidly under physiological conditions, presumably reflecting the natural abundance of LPL, that is required to liberate large quantities of FA from circulating post prandial CMs (the *in vivo* half-life of CMs is in the range of minutes (Park et al., 2001)).

The second step (i.e., drug release from the MG derivative), however, appears to be both critical and potentially limiting for systemic exposure (bioavailability) of active drug. Previous studies with MPA and TST suggest that parent drug release is dependent on the nature of the drug (and the functional group on the parent molecule that is used for prodrug conjugation). For example, MPA prodrugs conjugated via a carboxylic acid (Han et al., 2015) appear to provide more facile release compared to TST prodrugs conjugated via a secondary alcohol (Hu et al., 2016). In the current study, where BUP is conjugated to the TG backbone via a phenol, all prodrugs released BUP well upon incubation with LPL supplemented plasma (>50% over 3 h). This is in contrast to previous data with TST-C5-TG and TST-C5bMe-TG where release of TST under the same conditions was very low (Hu et al., 2016). The more complete *in vitro* BUP release from BUP-C4-TG and BUP-C5bMe-TG suggests that phenol containing prodrugs may provide for enhanced drug release in plasma, at least when compared to secondary alcohols such as TST, although more data is required to support this generalization. *In vitro* BUP release from even relatively simple C4 and C5bMe linkers was generally good, and addition of the CE and TML self immolative groups promoted drug release further such that *in vitro* BUP release from BUP-TML-C5bMe-TG (67%) and BUP-CE-C4-TG (75%) was slightly (but statistically significantly) higher compared to the non-SI analogues (BUP-C5bMe-TG (54%) and BUP-C4-TG (50%), respectively).

**FIGURE 8:**
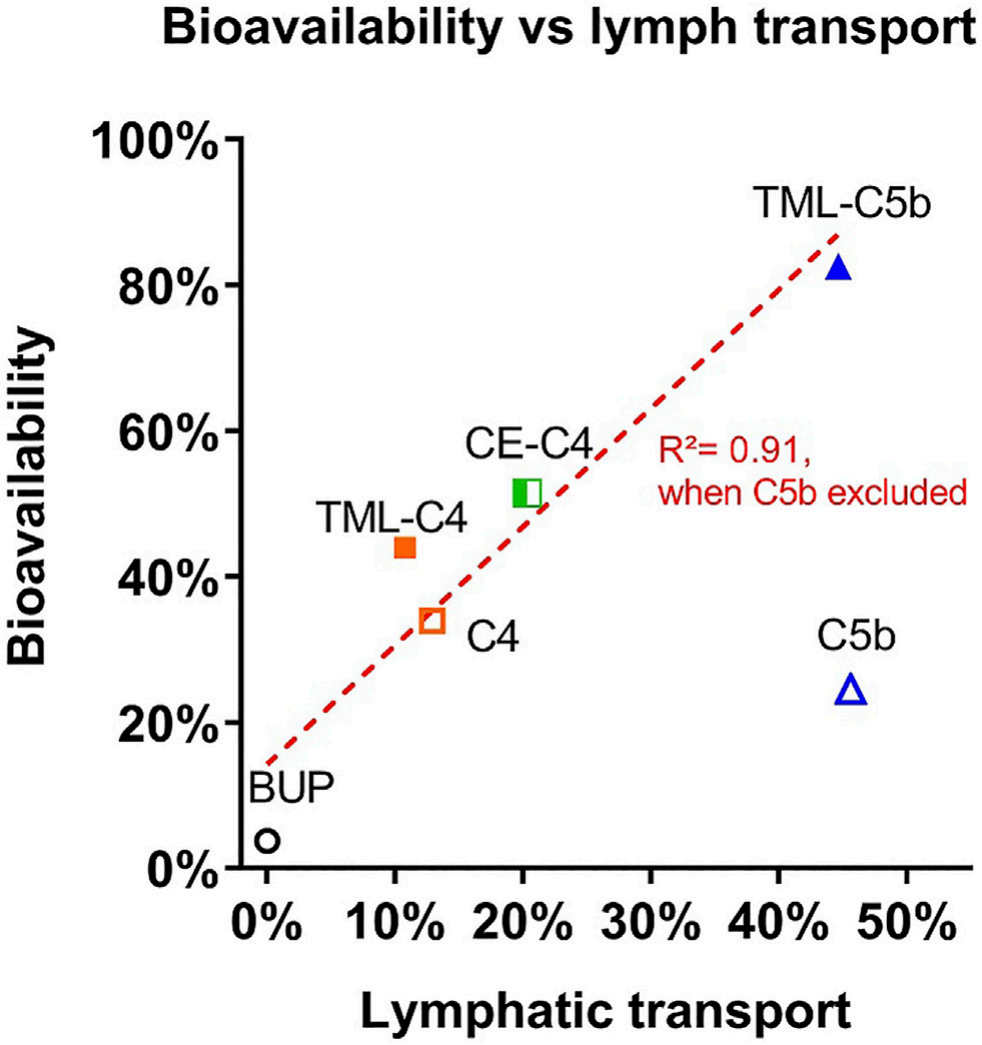
Comparison between oral bioavailability and lymphatic transport of the BUP prodrugs.

Interestingly, although the benefit of the SI linkers was relatively moderate *in vitro*, these differences were magnified *in vivo*, at least for the C5 linkers. Thus, BUP-C5bMe-TG and BUP-TML-C5bMe-TG showed similarly high lymphatic transport (∼45%), and the *in vitro* release of BUP in plasma was only slightly higher for the TML prodrug (67 vs. 54% BUP liberation), however, the oral BUP bioavailability from the TML C5bMe prodrug (83%) was more than 3-fold higher than the C5bMe prodrug (25%). These data suggest that the *in vitro* plasma release assay may not capture all the potential mechanisms of drug release *in vivo*. As a screening tool, however, and realizing that it under predicts *in vivo* release and is therefore conservative, it is probably fit for purpose. The advantage provided by the SI group was less apparent for the C4 prodrugs where the TML and CE derivatives did not display a clear advantage over the simple C4 linker in terms of bioavailability improvements. This likely reflects the inherently good release of BUP from all these constructs, such that plasma BUP exposure is instead limited by the relatively low lymphatic transport of the C4 variants (in turn a function of the lower GI stability of the C4 linker without the bMe “protection” in the C5 prodrugs). A correlation plot showing lymphatic transport versus bioavailability for all prodrugs (Figure 8), provides clarity of these issues and shows good correlation (*R* ^2^ = 0.91) for all except BUP-C5bMe-TG which had poorer release. The data suggest that for most of the prodrugs examined the extent of lymphatic transport was driving *in vivo* exposure. For BUP-C5bMe-TG, however, *in vivo* drug release was presumably limiting such that the addition of the TML linker was required to boost plasma release. In contrast for the C4 variants that lacked the bMe protecting group, GI stability was the dominant limitation reducing the extent of lymphatic transport.

Notably, the oral “bioavailability” of BUP after administration of BUP-TML-C5bMe-TG was extremely high (83%). This is not consistent with the fact that the extent of lymphatic transport was only 45% and the oral bioavailability of BUP alone is very low. As such bioavailability increases are expected to stem from increases in lymphatic transport. There are a number of potential explanations for this. Firstly, the pharmacokinetics of BUP are known to be non-linear (where unusually clearance and volume of distribution are higher at increasing dose). As such differences in the shape of the plasma profiles after oral and IV administration, and in particular high initial concentrations after IV administration, may lead to higher clearance and volume of distribution (and thus lower AUC) following IV administration, and over estimation of oral bioavailability (Gopal et al., 2002). In contrast, the bioavailability calculation employed here utilized truncated AUC data after oral administration due to enterohepatic recirculation, and this might be expected to lead to under (rather than over) estimation of BA. It also remains possible, that patterns of enterohepatic recycling may be different after oral and IV administration and this may complicate bioavailability estimates more generally. The clearance and volume of distribution of BUP after IV administration in an aqueous solution may also be different to that obtained after drainage of lymph containing re-esterified BUP-TML-C5b-TG into the systemic circulation and subsequent liberation of BUP. Finally, the extent of lymphatic transport was measured in anaesthetized rats in order to increase the success rate of the complex lymph cannulation surgery. It is therefore possible that anaesthesia underestimates the extent of lymphatic transport, although previous studies with other compounds (non-prodrugs) suggest that this is limited when using highly dispersed lipid-based formulations (Porter et al., 1996a; Porter et al., 1996b). There is therefore some uncertainty around absolute quantification of bioavailability. However, the same measure was used for all prodrugs and the relative trends are consistent. We are therefore confident that advantages in exposure are apparent after administration of the TG-mimetic prodrugs relative to BUP alone.

### BUP Prodrugs with Relatively High Molecular Weight Are Well Absorbed

One concern when embarking on this program to develop TG-mimetic prodrugs of BUP was the relatively high MW of BUP (468 Da). The TG-mimetic prodrug strategy does not add very significant molecular weight via the pro-moiety since the FA chains are removed *in situ* prior to absorption and then reattached in the enterocyte post-absorption. Nonetheless the linker and the glycerol backbone does add some mass and the MW of the MG intermediates (i.e., the species that are absorbed) ranged from 642 (C4-MG) to 874 (TML-C5bMe-MG). These are outside the typical rule-of-5 space and might therefore potentially limit intestinal permeability and bioavailability. In contrast, however, both lymphatic transport and bioavailability of BUP were high after administration of BUP-TML-C5bMe-TG and the T_max_ was quite short (1.3 h) suggesting efficient absorption of even the largest MG construct. Whether the lipophilicity of the MG derivative or the similarity in structure to endogenous MG plays a role in enhancing intestinal permeability is unknown at this stage (although there is limited evidence for the presence of functional MG uptake transporters in the small intestine) (Storch et al., 2008; Abumrad and Davidson 2012). Future studies will concentrate on expanding this work to a broader range of drugs and TG templates to better define the design criteria and limits for TG-mimetic prodrugs.

## Conclusion and Perspectives

The development of safe and effective analgesics has been an area of increasing research interest in the past few years in order to improve options available for the management of acute and chronic pain. Efforts to improve the design of analgesics have focused on 1) fundamental opioid receptor biology including exploration of biased activation of GPCR receptors to provide effective analgesia whilst reducing addiction potential (Manglik et al., 2016); 2) medicinal chemistry approaches to modify existing potent opioid molecules to provide safer analogues, e.g., from BUP to BU08028, which has reduced abuse liability in primates (Ding et al., 2016); and 3) formulation and co-dosing approaches to enhance the bioavailability of opioid analgesics (including BUP) at the same time as reducing abuse potential. The current study demonstrates the potential for a TG mimetic prodrug strategy to avoid the first-pass metabolism of BUP and markedly enhanced the oral bioavailability of BUP from 3.7% to up to ∼83% (realising the caveats around the accuracy of BA estimation). This greatly increases the potential to develop an orally administered BUP product for clinical use. In addition to the convenience brought about by oral administration, the prodrug approach may contribute to safer opioid use by reducing abuse potential when BUP is used as an analgesic and for opioid replacement therapy (as documented in the FDA’s guideline on Abuse-Deterrent Opioids (Abuse-Deterrent Opioids—Evaluation and Labeling 2015)). This suggestion is based on the realisation that firstly it is difficult to extract the BUP prodrug from an oral lipid formulation to allow eg IV injection and secondly that the need for conversion of the prodrug to parent BUP in the systemic circulation is likely to limit C_max_ and therefore the attainment of high peak concentrations.

The current work also provides insight into the design of lymph-directing prodrugs more generally. This should have application not only in enhancing oral BA via reductions in first pass metabolism (as exemplified in the current study) but also in the use of oral lymph directing glyceride prodrugs to facilitate targeted drug delivery to enhance treatment of diseases with possible lymphatic involvement such as HIV, cancer, organ failure, inflammatory and metabolic disease. Examples of the latter include 1) delivery of the antiviral lopinavir to HIV reservoirs in the mesenteric lymphatic system (Qin et al., 2021); 2) delivery of the anticancer agent docetaxel to specific tumor tissues, with reduced gastrointestinal toxicity (Tian et al., 2019); 3) delivery of a lymph directing prodrug of the immunomodulator mycophenolic acid to the mesenteric lymph nodes to enhance GI immunomodulation in an OVA-stimulated lymphocyte proliferation model (Kochappan et al., 2021); 4) delivery of a lymph directed prodrug of the NSAID celecoxib to the mesenteric lymphatics and the adipose tissue surrounding the lymphatics to improve insulin resistance in obese high-fat-diet fed animals (Cao et al., 2021) and the delivery of TG analogues of the natural antioxidant pterostilbene to adipose tissues (Mattarei et al., 2017) and 5) delivery of lipase inhibitors to the mesenteric lymphatics to neutralise pancreatic lipases, that are released into the lymphatics in acute and critical illness (Lee et al., 2021). Ongoing studies in our laboratories and others are exploring a range of approaches to enhance drug delivery and efficacy via the use of lymph-directing strategies.

## Data Availability

The original contributions presented in the study are included in the article/Supplementary Material, further inquiries can be directed to the corresponding authors.

## References

[B1] Abumrad N. A. Davidson N. O. (2012). Role of the Gut in Lipid Homeostasis. Physiol. Rev. 92, 1061–1085. 10.1152/physrev.00019.2011 22811425 PMC3589762

[B2] Abuse-Deterrent Opioids-Evaluation and Labeling (2015). Guidance for Industry Center for Drug Evaluation and Research. FDA. Available at: https://www.fda.gov/regulatory-information/search-fda-guidance-documents/abuse-deterrent-opioids-evaluation-and-labeling .

[B3] Amory J. K. Scriba G. K. Amory D. W. Bremner W. J. (2003). Oral Testosterone-Triglyceride Conjugate in Rabbits: Single-Dose Pharmacokinetics and Comparison with Oral Testosterone Undecanoate. J. Androl. 24, 716–720. 10.1002/j.1939-4640.2003.tb02732.x 12954663

[B4] Bala V. Rao S. Li P. Wang S. Prestidge C. A. (2016). Lipophilic Prodrugs of SN38: Synthesis and *In Vitro* Characterization toward Oral Chemotherapy. Mol. Pharm. 13 (13), 287–294. Jan 4. 10.1021/acs.molpharmaceut.5b00785 26623947

[B5] Bensadoun A. (1991). Lipoprotein Lipase. Annu. Rev. Nutr. 11, 217–237. 10.1146/annurev.nu.11.070191.001245 1892699

[B6] Cao E. Watt M. J. Nowell C. J. Quach T. Simpson J. S. De Melo Ferreira V. (2021). Mesenteric Lymphatic Dysfunction Promotes Insulin Resistance and Represents a Potential Treatment Target in Obesity. Nat. Metab. 3, 1175–1188. 10.1038/s42255-021-00457-w 34545251

[B7] Charman W. N. A. Stella V. J. (1986). Estimating the Maximal Potential for Intestinal Lymphatic Transport of Lipophilic Drug Molecules. Int. J. Pharmaceutics 34, 175–178. 10.1016/0378-5173(86)90027-x

[B8] Cifarelli V. Eichmann A. (2019). The Intestinal Lymphatic System: Functions and Metabolic Implications. Cell Mol. Gastroenterol. Hepatol. 7, 503–513. 10.1016/j.jcmgh.2018.12.002 30557701 PMC6396433

[B9] Coe M. A. Lofwall M. R. Walsh S. L. (2019). Buprenorphine Pharmacology Review: Update on Transmucosal and Long-Acting Formulations. J. Addict. Medmar-apr 13, 93–103. 10.1097/ADM.0000000000000457 PMC7442141 30531584

[B10] Daitch D. Daitch J. Novinson D. Frey M. Mitnick C. Pergolizzi J. Jr. (2014). Conversion from High-Dose Full-Opioid Agonists to Sublingual Buprenorphine Reduces Pain Scores and Improves Quality of Life for Chronic Pain Patients. Pain Med. 15, 2087–2094. 10.1111/pme.12520 25220043

[B11] Ding H. Czoty P. W. Kiguchi N. Cami-Kobeci G. Sukhtankar D. D. Nader M. A. (2016). A Novel Orvinol Analog, BU08028, as a Safe Opioid Analgesic Without Abuse Liability in Primates. Proc. Natl. Acad. Sci. U S A. 113, E5511–E5518. Sep 13. 10.1073/pnas.1605295113 27573832 PMC5027459

[B60] Elz A. S. Trevaskis N. L. Porter C. J. H. Bowen J. M. Prestidge C. A. (2022). Smart Design Approaches for Orally Administered Lipophilic Prodrugs to Promote Lymphatic Transport. J. Control Release 341, 676–701. 10.1016/j.jconrel.2021.12.003 34896450

[B12] Fihlman M. Hemmilä T. Hagelberg N. M. Backman J. T. Laitila J. Laine K. (2018). Voriconazole Greatly Increases the Exposure to Oral Buprenorphine. Eur. J. Clin. Pharmacol. 74, 1615–1622. 10.1007/s00228-018-2548-8 30167757

[B13] Franco V. Gershkovich P. Perucca E. Bialer M. (2020). The Interplay between Liver First-Pass Effect and Lymphatic Absorption of Cannabidiol and its Implications for Cannabidiol Oral Formulations. Clin. Pharmacokinet. 59, 1493–1500. 10.1007/s40262-020-00931-w 32785853

[B14] Garzon-Aburbeh A. Poupaert J. H. Claesen M. Dumont P. Atassi G. (1983). 1,3-dipalmitoylglycerol Ester of Chlorambucil as a Lymphotropic, Orally Administrable Antineoplastic Agent. J. Med. Chem. 26, 1200–1203. 10.1021/jm00362a021 6876088

[B15] Gopal S. Tzeng T. B. Cowan A. (2002). Characterization of the Pharmacokinetics of Buprenorphine and Norbuprenorphine in Rats after Intravenous Bolus Administration of Buprenorphine. Eur. J. Pharm. Sci. 15, 287–293. 10.1016/s0928-0987(02)00009-x 11923061

[B16] Grisham G. Gutierrez L. A. Nelson M. T. Mikals K. Powell A. (2019). Contact Hypersensitivity Stomatitis in Response to Suboxone Use: A Case Report. Oral Maxillofac. Surg. Cases 5, 100122. 10.1016/j.omsc.2019.100122

[B17] Hale M. Garofoli M. Raffa R. B. (2021). Benefit-Risk Analysis of Buprenorphine for Pain Management. J. Pain Res. 14, 1359–1369. 10.2147/JPR.S305146 34079354 PMC8163969

[B18] Han S. Hu L. Quach T. Simpson J. S. Trevaskis N. L. Porter C. J. (2015). Profiling the Role of Deacylation-Reacylation in the Lymphatic Transport of a Triglyceride-Mimetic Prodrug. Pharm. Res. 32, 1830–1844. 10.1007/s11095-014-1579-9 25446770

[B19] Han S. Quach T. Hu L. Wahab A. Charman W. N. Stella V. J. (2014). Targeted Delivery of a Model Immunomodulator to the Lymphatic System: Comparison of Alkyl Ester versus Triglyceride Mimetic Lipid Prodrug Strategies. J. Control. Release 177, 1–10. Jan 5. 10.1016/j.jconrel.2013.12.031 24398334

[B20] Hu L. Quach T. Han S. Lim S. F. Yadav P. Senyschyn D. (2016). Glyceride-Mimetic Prodrugs Incorporating Self-Immolative Spacers Promote Lymphatic Transport, Avoid First-Pass Metabolism, and Enhance Oral Bioavailability. Angew. Chem. Int. Ed. Engl. 55, 13700–13705. 10.1002/anie.201604207 27482655

[B21] Irby D. Du C. Li F. (2017). Lipid-Drug Conjugate for Enhancing Drug Delivery. Mol. Pharm. 14, 1325–1338. 10.1021/acs.molpharmaceut.6b01027 28080053 PMC5477224

[B22] Johnson B. M. Chen W. Borchardt R. T. Charman W. N. Porter C. J. (2003). A Kinetic Evaluation of the Absorption, Efflux, and Metabolism of Verapamil in the Autoperfused Rat Jejunum. J. Pharmacol. Exp. Ther. 305, 151–158. Apr. 10.1124/jpet.102.045328 12649363

[B23] Johnson R. E. Fudala P. J. Payne R. (2005). Buprenorphine: Considerations for Pain Management. J. Pain Symptom. Managemar. 29, 297–326. 10.1016/j.jpainsymman.2004.07.005 15781180

[B24] Joshi A. Halquist M. Konsoula Z. Liu Y. Jones J. P. Heidbreder C. (2017). Improving the Oral Bioavailability of Buprenorphine: an *In-Vivo* Proof of Concept. J. Pharm. Pharmacol. 69, 23–31. 10.1111/jphp.12652 27781278

[B25] Kochappan R. Cao E. Han S. Hu L. Quach T. Senyschyn D. (2021). Targeted Delivery of Mycophenolic Acid to the Mesenteric Lymph Node Using a Triglyceride Mimetic Prodrug Approach Enhances Gut-specific Immunomodulation in Mice. J. Control. Release 332, 636–651. 10.1016/j.jconrel.2021.02.008 33609620

[B26] Kress H. G. (2009). Clinical Update on the Pharmacology, Efficacy and Safety of Transdermal Buprenorphine. Eur. J. Pain 13, 219–230. Mar. 10.1016/j.ejpain.2008.04.011 18567516

[B27] Lee G. Han S. Lu Z. Hong J. Phillips A. R. J. Windsor J. A. (2021). Intestinal Delivery in a Long-Chain Fatty Acid Formulation Enables Lymphatic Transport and Systemic Exposure of Orlistat. Int. J. Pharm. 596, 120247. 10.1016/j.ijpharm.2021.120247 33486039

[B28] Likar R. Kayser H. Sittl R. (2006). Long-term Management of Chronic Pain with Transdermal Buprenorphine: A Multicenter, Open-Label, Follow-Up Study in Patients from Three Short-Term Clinical Trials. Clin. Ther. 28, 943–952. 10.1016/j.clinthera.2006.06.012 16860176

[B29] Maharao N. V. Joshi A. A. Gerk P. M. (2017). Inhibition of Glucuronidation and Oxidative Metabolism of Buprenorphine Using GRAS Compounds or Dietary Constituents/Supplements: *In Vitro* Proof of Concept. Biopharm. Drug Dispos. 38, 139–154. 10.1002/bdd.2050 27925249

[B30] Manglik A. Lin H. Aryal D. K. McCorvy J. D. Dengler D. Corder G. (2016). Structure-Based Discovery of Opioid Analgesics with Reduced Side Effects. Nature 537, 185–190. 10.1038/nature19112 27533032 PMC5161585

[B31] Mannila A. Morizzi J. Nguyen T. T. Charman S. A. McIntosh M. P. Shackleford D. M. (2012). Probing a Potential *In Vivo* Drug-Excipient Interaction: Temporal Effects of a Modified β-Cyclodextrin on the Intravenous Pharmacokinetics of a Model High-Affinity Drug Ligand. J. Pharm. Sci. 101, 3381–3389. 10.1002/jps.23177 22549698

[B32] Markovic M. Ben-Shabat S. Keinan S. Aponick A. Zimmermann E. M. Dahan A. (2019). Lipidic Prodrug Approach for Improved Oral Drug Delivery and Therapy. Med. Res. Rev. 39, 579–607. 10.1002/med.21533 30320896

[B61] Mattarei A. Rossa A. Bombardelli V. Azzolini M. La Spina M. Paradisi C. (2017). Novel Lipid-Mimetic Prodrugs Delivering Active Compounds to Adipose Tissue. European J. Med. Chem. 135, 77–88. 28433778 10.1016/j.ejmech.2017.04.034

[B33] Mead J. R. Irvine S. A. Ramji D. P. (2002). Lipoprotein Lipase: Structure, Function, Regulation, and Role in Disease. J. Mol. Med. (Berl) 80, 753–769. 10.1007/s00109-002-0384-9 12483461

[B34] Mercadante S. Casuccio A. Tirelli W. Giarratano A. (2009). Equipotent Doses to Switch from High Doses of Opioids to Transdermal Buprenorphine. Support Care Cancer 17, 715–718. June. 10.1007/s00520-008-0546-6 19104845

[B35] Merkel M. Eckel R. H. Goldberg I. J. (2002). Lipoprotein Lipase: Genetics, Lipid Uptake, and Regulation. J. Lipid Res. 43, 1997–2006. 10.1194/jlr.r200015-jlr200 12454259

[B36] Ohtani M. Kotaki H. Uchino K. Sawada Y. Iga T. (1994). Pharmacokinetic Analysis of Enterohepatic Circulation of Buprenorphine and its Active Metabolite, Norbuprenorphine, in Rats. Drug Metab. Dispos. 22, 2–7. 8149883

[B37] Park Y. Damron B. D. Miles J. M. Harris W. S. (2001). Measurement of Human Chylomicron Triglyceride Clearance with a Labeled Commercial Lipid Emulsion. Lipids 36, 115–120. 10.1007/s11745-001-0696-6 11269690

[B38] Porter C. J. Charman S. A. Charman W. N. (1996a). Lymphatic Transport of Halofantrine in the Triple-Cannulated Anesthetized Rat Model: Effect of Lipid Vehicle Dispersion. J. Pharm. Sci. 85, 351–356. 10.1021/js950221g 8901067

[B39] Porter C. J. Charman S. A. Humberstone A. J. Charman W. N. (1996b). Lymphatic Transport of Halofantrine in the Conscious Rat when Administered as Either the Free Base or the Hydrochloride Salt: Effect of Lipid Class and Lipid Vehicle Dispersion. J. Pharm. Sci. 85, 357–361. 10.1021/js9502229 8901068

[B40] Porter C. J. Charman W. N. (2001). Intestinal Lymphatic Drug Transport: an Update. Adv. Drug Deliv. Rev. 50, 61–80. Aug 23. 10.1016/s0169-409x(01)00151-x 11489334

[B41] Prommer E. (2015). Buprenorphine for Cancer Pain. Am. J. Hosp. Palliat. Care 32, 881–889. 10.1177/1049909114547227 25163678

[B42] Qin C. Chu Y. Feng W. Fromont C. He S. Ali J. (2021). Targeted Delivery of Lopinavir to HIV Reservoirs in the Mesenteric Lymphatic System by Lipophilic Ester Prodrug Approach. J. Controlled Release 329, 1077–1089. 10.1016/j.jconrel.2020.10.036 33091528

[B43] Scriba G. K. (1995). Synthesis and *In Vitro* Degradation of Testosterone-Lipid Conjugates. Arch. Pharm. (Weinheim) 328, 271–276. 10.1002/ardp.19953280313 7763143

[B44] Serpell M. Tripathi S. Scherzinger S. Rojas-Farreras S. Oksche A. Wilson M. (2016). Assessment of Transdermal Buprenorphine Patches for the Treatment of Chronic Pain in a UK Observational Study. Patient 9, 35–46. 10.1007/s40271-015-0151-y 26547914 PMC4720699

[B45] Shackleford D. M. Faassen W. A. Houwing N. Lass H. Edwards G. A. Porter C. J. (2003). Contribution of Lymphatically Transported Testosterone Undecanoate to the Systemic Exposure of Testosterone after Oral Administration of Two Andriol Formulations in Conscious Lymph Duct-Cannulated Dogs. J. Pharmacol. Exp. Ther. 306, 925–933. 10.1124/jpet.103.052522 12807999

[B46] Shi Y. Cheng D. (2009). Beyond Triglyceride Synthesis: the Dynamic Functional Roles of MGAT and DGAT Enzymes in Energy Metabolism. Am. J. Physiol. Endocrinol. Metab. 297, E10–E18. 10.1152/ajpendo.90949.2008 19116371 PMC3735925

[B47] Storch J. Zhou Y. X. Lagakos W. S. (2008). Metabolism of Apical versus Basolateral Sn-2-Monoacylglycerol and Fatty Acids in Rodent Small Intestine. J. Lipid Res. 49, 1762–1769. 10.1194/jlr.M800116-JLR200 18421071 PMC2444011

[B48] Strang J. Knight A. Baillie S. Reed K. Bogdanowicz K. Bell J. (2018). Norbuprenorphine and Respiratory Depression: Exploratory Analyses with New Lyophilized Buprenorphine and Sublingual Buprenorphine. Int. J. Clin. Pharmacol. Ther. 56, 81–85. 10.5414/CP203118 29231163

[B49] Sugihara J. Furuuchi S. Ando H. Takashima K. Harigaya S. (1988). Studies on Intestinal Lymphatic Absorption of Drugs. II. Glyceride Prodrugs for Improving Lymphatic Absorption of Naproxen and Nicotinic Acid. J. Pharmacobiodyn 11, 555–562. 10.1248/bpb1978.11.555 3236213

[B50] Suzuki J. Mittal L. Woo S. B. (2013). Sublingual Buprenorphine and Dental Problems: A Case Series. Prim. Care Companion CNS Disord. 15. 10.4088/PCC.13l01533 PMC3907320 24511440

[B51] Täuber U. Schröder K. Düsterberg B. Matthes H. (1986a). Absolute Bioavailability of Testosterone After Oral Administration of Testosterone-Undecanoate and Testosterone. Eur. J. Drug Metab. Pharmacokinet. 11, 145–149. 10.1007/BF03189840 3770015

[B52] Täuber U. Schröder K. Düsterberg B. Matthes H. (1986b). Absolute Bioavailability of Testosterone After Oral Administration of Testosterone-Undecanoate and Testosterone. Eur. J. Drug Metab. Pharmacokinet. 11, 145–149. 10.1007/BF03189840 3770015

[B53] Taylor R. Raffa R. B. Pergolizzi J. V. (2013). “Buprenorphine Metabolism and Drug-Drug Interactions,” in Handbook of Methadone Prescribing and Buprenorphine Therapy (New York, NY: Springer), 183–200. 10.1007/978-1-4614-6974-2_13

[B54] Tian C. Guo J. Wang G. Sun B. Na K. Zhang X. (2019). Efficient Intestinal Digestion and on Site Tumor-Bioactivation Are the Two Important Determinants for Chylomicron-Mediated Lymph-Targeting Triglyceride-Mimetic Docetaxel Oral Prodrugs. Adv. Sci. (Weinh) 6, 1901810. 10.1002/advs.201901810 31871861 PMC6918103

[B55] Trevaskis N. L. Charman W. N. Porter C. J. (2008). Lipid-based Delivery Systems and Intestinal Lymphatic Drug Transport: A Mechanistic Update. Adv. Drug Deliv. Rev. 60, 702–716. Mar. 17. 10.1016/j.addr.2007.09.007 18155316 PMC7103284

[B56] Trevaskis N. L. Hu L. Caliph S. M. Han S. Porter C. J. H. (2015). The Mesenteric Lymph Duct Cannulated Rat Model: Application to the Assessment of Intestinal Lymphatic Drug Transport. J. Visualized Experiments : JoVE. 10.3791/52389 PMC4401200 25866901

[B62] Wang X. Zhang C. Han N. Luo J. Zhang S. Wang C. (2021). Triglyceride-Mimetic Prodrugs of Scutellarin Enhance Oral Bioavailability by Promoting Intestinal Lymphatic Transport and Avoiding First-Pass Metabolism. Drug Deliv. 28, 1664–1672. 34338567 10.1080/10717544.2021.1960928 PMC8330727

[B57] Wolff R. F. Aune D. Truyers C. Hernandez A. V. Misso K. Riemsma R. (2012). Systematic Review of Efficacy and Safety of Buprenorphine versus Fentanyl or Morphine in Patients with Chronic Moderate to Severe Pain. Curr. Med. Res. Opin. 28, 833–845. 10.1185/03007995.2012.678938 22443154

[B58] Yáñez J. A. Wang S. W. Knemeyer I. W. Wirth M. A. Alton K. B. (2011). Intestinal Lymphatic Transport for Drug Delivery. Adv. Drug Deliv. Rev. 63, 923–942. Sep 10. 10.1016/j.addr.2011.05.019 21689702 PMC7126116

[B59] Zhang F. Zarkada G. Han J. Li J. Dubrac A. Ola R. (2018). Lacteal junction Zippering Protects Against Diet-Induced Obesity. Science 361, 599–603. 10.1126/science.aap9331 30093598 PMC6317738

