# Corrigendum: Triglyceride-mimetic prodrugs of buprenorphine enhance oral bioavailability via promotion of lymphatic transport

**DOI:** 10.3389/fphar.2022.1014363

**Published:** 2022-09-05

**Authors:** Tim Quach, Luojuan Hu, Sifei Han, Shea F. Lim, Danielle Senyschyn, Preeti Yadav, Natalie L. Trevaskis, Jamie S. Simpson, Christopher J. H. Porter

**Affiliations:** ^1^ Medicinal Chemistry, Monash Institute of Pharmaceutical Sciences, Monash University, Parkville, VIC, Australia; ^2^ Drug Delivery, Disposition and Dynamics, Monash Institute of Pharmaceutical Sciences, Monash University, Parkville, VIC, Australia

**Keywords:** lymphatic transport, prodrug, triglyceride mimetic, buprenorphine, first-pass metabolism, oral bioavailability, opioid analgesics

In the published article, an author name was incorrectly written as Trevaksis. The correct spelling is Trevaskis.

Note this error is repeated in the **main heading** and in the citation information on page 1. It is also incorrect in the Copyright statement.

The authors apologize for this error and state that this does not change the scientific conclusions of the article in any way. The original article has been updated.

