# Supplementary material for: Triglyceride-Mimetic Prodrugs of Buprenorphine Enhance Oral Bioavailability via Promotion of Lymphatic Transport
Source: Front Pharmacol. 2022 Apr 12;13:879660. doi: 10.3389/fphar.2022.879660 (PMC9039622; doi:10.3389/fphar.2022.879660)
Supplement: Supplementary file 1 [file Table1.DOCX]

Supplementary Material

**Triglyceride-mimetic Prodrugs of Buprenorphine Enhance Oral Bioavailability via Promotion of Lymphatic Transport**

**Tim Quach^1,†^, Luojuan Hu^2,†^, Sifei Han^2,^*, Shea F. Lim^1^, Danielle Senyschyn^2^, Preeti Yadav^2^, Natalie L. Trevaksis^2^, Jamie S. Simpson^1^, Christopher J.H. Porter^2,^***

^1^Medicinal Chemistry, Monash Institute of Pharmaceutical Sciences, Monash University, Parkville, VIC 3052, Australia

^2^Drug Delivery, Disposition and Dynamics, Monash Institute of Pharmaceutical Sciences, Monash University, Parkville, VIC 3052, Australia

† These authors have contributed equally to this work

*** Correspondence:**Sifei Han ([sifei.han@monash.edu](mailto:sifei.han@monash.edu)) and Christopher Porter ([chris.porter@monash.edu](mailto:chris.porter@monash.edu))

**1. Synthesis and characterisation of BUP prodrugs**

**1.1 General experimental section**

All commercially available chemicals and solvents were purchased from either Sigma-Aldrich, Acros, or Merck, with the exception of palmitic acid (Ajax Chemicals, Sydney, Australia). For anhydrous reactions, HPLC-grade CH_2_Cl_2_, THF and DMF were dried using an MBraun solvent purification system (SPS-800) according to manufacturer’s instructions. For small-scale chromatography, hexanes and ethyl acetate were distilled before use. All other chemicals and solvents were reagent grade and used as received.

Thin layer chromatography (TLC) was used to monitor reaction progress and was performed on Merck Silica Gel 60 F254 plates. TLC plates were visualised under UV light at 254 nm and/or with the aid of staining with either phosphomolybdic acid (PMA) or potassium permanganate. Column chromatography was achieved using Davisil silica gel LC60A (40-63 micron).

All ^1^H and ^13^C NMR spectra were recorded on a Bruker Avance 400 MHz NMR spectrometer. Data acquisition and processing was managed using Topspin/ICONNMR NMR software package and plotting was managed using MestReNova v6.0.2-475. All spectra were run in deuterochloroform, unless otherwise stated. All chemical shifts were measured in parts per million (ppm) referenced to an internal standard of residual proteosolvent (CHCl_3_) at δ 7.26 ppm for ^1^H NMR and δ 77.16 ppm for ^13^C NMR. Spectroscopic resonances were assigned using the following abbreviations: s, singlet; d, doublet; t, triplet; q, quartet; m, multiplet.

High Resolution Mass Spectrometry (HRMS) analyses were collected on an Agilent 6224 time-of-flight (TOF) LC/MS Mass Spectrometer coupled to an Agilent 1290 Infinity (Agilent, Palo Alto, CA). All data were acquired and reference mass corrected via a dual‐spray electrospray ionisation (ESI) source. Each scan or data point on the Total Ion Chromatogram (TIC) is an average of 13,700 transients, producing a spectrum every second. Mass spectra were created by averaging the scans across each peak and background subtracted against the first 10 seconds of the TIC. Acquisition was performed using the Agilent Mass Hunter Data Acquisition software version B.05.00 Build 5.0.5042.2 and analysis was performed using Mass Hunter Qualitative Analysis version B.05.00 Build 5.0.519.13.

**1.2 Synthesis of BUP-C4-TG (4)**

*Synthesis of* ***2*:** 4-(Dimethylamino)pyridine (DMAP, 15.5 mg, 0.127 mmol) was added to a solution of 1,3-diglyceride **1^1^** (72.2 mg, 0.127 mmol) and succinic anhydride (25.4 mg, 0.254 mmol) in pyridine/THF/CH_2_Cl_2_ (0.5 mL each) and the mixture stirred at rt for 17 hours. An extra portion of succinic anhydride (25.4 mg, 0.254 mmol) and DMAP (15.5 mg, 0.127 mmol) was added and the solution heated at 40 °C for a further 22 hours. The reaction was diluted with ethyl acetate (25 mL), washed with 1 M HCl (20 mL) and brine (2 × 30 mL), dried (MgSO_4_) and concentrated under reduced pressure to give the crude product. Silica gel chromatography (15% to 25% ethyl acetate/hexanes) gave acid-TG **2** (77.0 mg, 91%) as a colourless solid; ^1^H NMR (400 MHz, CDCl_3_) δ 5.27 (m, 1H), 4.30 (dd, *J* = 12.0, 4.3 Hz, 2H), 4.15 (dd, *J* = 12.0, 5.8 Hz, 2H), 2.72 – 2.61 (m, 4H), 2.31 (t, *J* = 7.6 Hz, 4H), 1.67 – 1.54 (m, 4H), 1.36 – 1.19 (m, 48H), 0.88 (t, *J* = 6.9 Hz, 6H); ^13^C NMR (101 MHz, CDCl_3_) δ 176.9 (C), 173.5 (2C; C), 171.4 (C), 69.8 (CH), 62.0 (2C; CH_2_), 34.2 (2C; CH_2_), 32.1 (2C; CH_2_), 29.84 (6C; CH_2_), 29.81 (4C; CH_2_), 29.77 (2C; CH_2_), 29.6 (2C; CH_2_), 29.5 (2C; CH_2_), 29.4 (2C; CH_2_), 29.3 (2C; CH_2_), 29.0 (CH_2_), 28.8 (CH_2_), 25.0 (2C; CH_2_), 22.8 (2C; CH_2_), 14.3 (2C; CH_3_).

*Synthesis of* **4:** 4-(Dimethylamino)pyridine (DMAP, 2.0 mg, 16.0 μmol) and EDC•HCl (7.7 mg, 40.0 μmol) were added to a solution of acid-TG **2** (13.9 mg, 20.8 μmol) and buprenorphine (**3**) (7.5 mg, 16.0 μmol) in CH_2_Cl_2_ (0.8 mL) and the mixture stirred at rt for two hours. The reaction was diluted with CH_2_Cl_2_ (5 mL), silica gel was added and the solvent removed under reduced pressure. Purification by silica gel chromatography (10% to 15% ethyl acetate/hexanes with 0.5% Et_3_N) gave BUP prodrug **4** (15.6 mg, 87%) as a colourless oil; ^1^H NMR (401 MHz, CDCl_3_) δ 6.78 (d, *J* = 8.1 Hz, 1H), 6.58 (d, *J* = 8.1 Hz, 1H), 5.89 (s, 1H), 5.26 (m, 1H), 4.42 (d, *J* = 1.6 Hz, 1H), 4.29 (dd, *J* = 11.9, 4.4 Hz, 2H), 4.19 – 4.12 (m, 2H), 3.45 (s, 3H), 3.05 – 2.97 (m, 2H), 2.93 – 2.83 (m, 3H), 2.70 (t, *J* = 6.9 Hz, 2H), 2.62 (dd, *J* = 11.8, 4.9 Hz, 1H), 2.37 – 2.20 (m, 8H), 2.11 (t, *J* = 9.8 Hz, 1H), 1.96 (m, 1H), 1.91 – 1.75 (m, 2H), 1.70 (dd, *J* = 13.0, 2.4 Hz, 1H), 1.65 – 1.53 (m, 4H), 1.35 (s, 3H), 1.37 – 1.16 (m, 49H), 1.06 (m, 1H), 1.03 (s, 9H), 0.88 (t, *J* = 6.9 Hz, 6H), 0.80 (m, 1H), 0.67 (m, 1H), 0.54 – 0.42 (m, 2H), 0.15 – 0.08 (m, 2H).

**1.3 Synthesis of BUP-C5bMe-TG (6)**

4-(Dimethylamino)pyridine (DMAP, 2.1 mg, 17.1 μmol) and EDC•HCl (8.2 mg, 42.8 μmol) were added to a solution of acid-TG **5^2^** (15.5 mg, 22.2 μmol) and buprenorphine (**3**) (8.0 mg, 17.1 μmol) in CH_2_Cl_2_ (0.8 mL) and the mixture stirred at rt for 18 hours. The reaction was diluted with CH_2_Cl_2_ (10 mL), silica gel was added and the solvent removed under reduced pressure. Purification by silica gel chromatography (12.5% to 15% ethyl acetate/hexanes) gave BUP prodrug **6** (17.0 mg, 87%) as a colourless oil; ^1^H NMR (401 MHz, CDCl_3_) δ 6.76 (d, *J* = 7.6 Hz, 1H), 6.59 (d, *J* = 8.1 Hz, 1H), 5.88 (s, 1H), 5.28 (m, 1H), 4.42 (s, 1H), 4.34 – 4.26 (m, 2H), 4.13 (dd, *J* = 11.9, 6.0 Hz, 2H), 3.45 (s, 3H), 3.06 – 2.98 (m, 2H), 2.88 (m, 1H), 2.67 – 2.59 (m, 2H), 2.57 – 2.41 (m, 3H), 2.39 – 2.21 (m, 9H), 2.11 (m, 1H), 2.02 – 1.78 (m, 3H), 1.71 (m, 1H), 1.65 – 1.54 (m, 4H), 1.35 (s, 3H), 1.38 – 1.19 (m, 49H), 1.102/1.091 (each d, *J* = 6.4 Hz, 3H), 1.04 (m, 1H), 1.03 (s, 9H), 0.87 (t, *J* = 6.9 Hz, 8H), 0.79 (m, 1H), 0.70 (m, 1H), 0.55 – 0.42 (m, 2H), 0.15 – 0.08 (m, 2H); ^13^C NMR (101 MHz, CDCl_3_) δ 173.4 (2C; C), 171.4 (C), 170.1 (C), 149.9 (C), 134.6 (C), 133.9 (C), 131.3 (C), 122.1 (CH), 119.4 (CH), 98.2 (CH), 80.9 (C), 79.4 (C), 69.3 (CH), 62.2 (2C; CH_2_), 59.7 (CH_2_), 58.3 (CH), 52.7 (CH_3_), 46.4 (C), 44.4 (CH), 43.7 (CH_2_), 40.69/40.60 (CH_2_), 40.5 (C), 40.36/40.32 (CH_2_), 36.1 (C), 35.6 (CH_2_), 34.1 (2C; CH_2_), 33.5 (CH_2_), 32.1 (2C; CH_2_), 29.9 (CH_2_), 29.85 (6C; CH_2_), 29.81 (4C; CH_2_), 29.77 (2C; CH_2_), 29.6 (2C; CH_2_), 29.5 (2C; CH_2_), 29.4 (2C; CH_2_), 29.3 (2C; CH_2_), 27.68/27.64 (CH), 26.6 (3C; CH_3_), 25.0 (2C; CH_2_), 23.5 (CH_2_), 22.8 (2C; CH_2_), 20.1 (CH_3_), 19.62/19.60 (CH_3_), 17.7 (CH_2_), 14.3 (2C; CH_3_), 9.6 (CH), 4.3 (CH_2_), 3.4 (CH_2_).

**1.4 Synthesis of BUP-CE-C4-TG (8)**

*Synthesis of* **7:** 4-(Dimethylamino)pyridine (DMAP, 3.1 mg, 25.7 μmol) and EDC•HCl (12.3 mg, 64.2 μmol) were added to a solution of buprenorphine (**3**) (12.0 mg, 25.7 μmol) and 5-bromovaleric acid (7.4 mg, 41.1 μmol) in CH_2_Cl_2_ (1.2 mL) and the mixture stirred at rt for two hours. The reaction was diluted with CH_2_Cl_2_ (5 mL), silica gel was added and the mixture concentrated under reduced pressure. Purification by silica gel chromatography (12.5% ethyl acetate/hexanes) gave BUP ester **7** (15.0 mg, 93%) as a colourless oil; ^1^H NMR (401 MHz, CDCl_3_) δ 6.77 (d, *J* = 8.1 Hz, 1H), 6.59 (d, *J* = 8.1 Hz, 1H), 5.88 (s, 1H), 4.43 (d, *J* = 1.6 Hz, 1H), 3.46 (s, 3H), 3.42 (t, *J* = 6.5 Hz, 2H), 3.02 (d, *J* = 14.4 Hz, 1H), 2.99 (s, 1H), 2.88 (m, 1H), 2.62 (dd, *J* = 12.2, 5.2 Hz, 1H), 2.57 (t, *J* = 7.1 Hz, 2H), 2.38 – 2.22 (m, 4H), 2.12 (t, *J* = 9.8 Hz, 1H), 2.02 – 1.92 (m, 3H), 1.91 – 1.77 (m, 4H), 1.71 (dd, *J* = 12.9, 2.3 Hz, 1H), 1.35 (s, 3H), 1.30 (m, 1H), 1.05 (m, 1H), 1.03 (s, 9H), 0.80 (m, 1H), 0.68 (m, 1H), 0.56 – 0.43 (m, 2H), 0.15 – 0.07 (m, 2H).

*Synthesis of* **8:** 1,8-Diazabicyclo[5.4.0]undec-7-ene (DBU) (2.8 μL, 19.0 μmol) was added to a suspension of acid-TG **2** (8.0 mg, 11.9 μmol) and bromide **7** (7.5 mg, 11.9 μmol) in toluene (0.8 mL) and the mixture heated at reflux for three hours. The reaction was cooled to rt, then diluted with ethyl acetate (25 mL). The organic phase was washed with water (20 mL) and brine (20 mL), dried (MgSO_4_) and concentrated under reduced pressure to give the crude product. Silica gel chromatography (10% to 20% ethyl acetate/hexane) gave BUP-CE prodrug **8** (1.6 mg, 11%) as a colourless solid; ^1^H NMR (401 MHz, CDCl_3_) δ 6.77 (d, *J* = 8.1 Hz, 1H), 6.59 (d, *J* = 8.1 Hz, 1H), 5.88 (s, 1H), 5.26 (m, 1H), 4.42 (d, *J* = 1.6 Hz, 1H), 4.29 (dd, *J* = 11.9, 4.4 Hz, 2H), 4.15 (dd, *J* = 12.0, 5.8 Hz, 2H), 4.11 (t, *J* = 6.0 Hz, 2H), 3.45 (s, 3H), 3.02 (d, *J* = 13.5 Hz, 1H), 2.99 (s, 1H), 2.88 (m, 1H), 2.67 – 2.54 (m, 7H), 2.38 – 2.22 (m, 8H), 2.11 (t, *J* = 10.1 Hz, 1H), 1.97 (td, *J* = 12.7, 5.6 Hz, 1H), 1.91 – 1.84 (m, 2H), 1.82 – 1.68 (m, 5H), 1.65 – 1.57 (m, 4H), 1.35 (s, 3H), 1.35 – 1.19 (m, 49H), 1.05 (m, 1H), 1.03 (s, 9H), 0.88 (t, *J* = 6.9 Hz, 6H), 0.84 – 0.76 (m, 1H), 0.75 – 0.63 (m, 1H), 0.55 – 0.43 (m, 2H), 0.17 – 0.08 (m, 2H).

**1.5 Synthesis of BUP-TML-C4-TG (14)**

*Synthesis of* **10:** 4-(Dimethylamino)pyridine (DMAP, 18.3 mg, 0.149 mmol) and EDC•HCl (71.6 mg, 0.374 mmol) were added to a solution of acid-TG **2** (100 mg, 0.149 mmol) and phenol **9^3^** (53.0 mg, 0.164 mmol) in CH_2_Cl_2_ (4 mL) and the mixture stirred at rt for 19 hours. The reaction was diluted with CH_2_Cl_2_ (5 mL), silica gel was added and the mixture concentrated under reduced pressure. Purification by silica gel chromatography (3% to 7.5% ethyl acetate/hexanes) gave TML-TG **10** (84.6 mg, 58%) as a colourless oil; ^1^H NMR (400 MHz, CDCl_3_) δ 6.80 (d, *J* = 2.0 Hz, 1H), 6.55 (d, *J* = 1.9 Hz, 1H), 5.29 (m, 1H), 4.31 (dd, *J* = 11.9, 4.4 Hz, 2H), 4.16 (dd, *J* = 12.0, 5.8 Hz, 2H), 3.51 – 3.44 (m, 2H), 2.85 (t, *J* = 6.9 Hz, 2H), 2.75 (t, *J* = 6.9 Hz, 2H), 2.51 (s, 3H), 2.30 (t, *J* = 7.6 Hz, 4H), 2.22 (s, 3H), 2.06 – 1.99 (m, 2H), 1.65 – 1.56 (m, 4H), 1.46 (s, 6H), 1.37 – 1.20 (m, 48H), 0.88 (t, *J* = 6.9 Hz, 6H), 0.84 (s, 9H), -0.03 (s, 6H); ^13^C NMR (101 MHz, CDCl_3_) δ 173.4 (2C; C), 171.5 (C), 171.3 (C), 149.7 (C), 138.5 (C), 136.1 (C), 134.1 (C), 132.5 (CH), 123.1 (CH), 69.8 (CH), 62.0 (2C; CH_2_), 60.9 (CH_2_), 46.1 (CH_2_), 39.2 (C), 34.1 (2C; CH_2_), 32.1 (2C; CH_2_), 31.9 (2C; CH_3_), 29.9 (CH_2_), 29.83 (6C; CH_2_), 29.79 (4C; CH_2_), 29.75 (2C; CH_2_), 29.6 (2C; CH_2_), 29.5 (2C; CH_2_), 29.4 (2C; CH_2_), 29.2 (2C; CH_2_), 29.0 (CH_2_), 26.1 (3C; CH_3_), 25.4 (CH_3_), 25.0 (2C; CH_2_), 22.8 (2C; CH_2_), 20.3 (CH_3_), 18.3 (C), 14.3 (2C; CH_3_), -5.21 (2C; CH_3_); ESI-HRMS: calcd. for C_58_H_105_O_9_Si [M + H^+^] 973.7522; found 973.7515.

*Synthesis of* **11:** 10-Camphorsulfonic acid (3.0 mg, 12.9 μmol) was added to TBS ether **10** (83.7 mg, 86.0 μmol) in CH_2_Cl_2_ (1 mL) and MeOH (1 mL) and the mixture stirred at rt for one hour. The reaction was diluted with CH_2_Cl_2_ (20 mL) and the organic phase washed with sat. aq. NaHCO_3_ and brine (20 mL each), dried (MgSO_4_) and concentrated under reduced pressure to give the crude product. Purification by silica gel chromatography (15% to 25% ethyl acetate/hexanes) gave alcohol **11** (59.9 mg, 81%) as a colourless oil; ^1^H NMR (400 MHz, CDCl_3_) δ 6.81 (d, *J* = 2.0 Hz, 1H), 6.56 (d, *J* = 1.4 Hz, 1H), 5.28 (m, 1H), 4.30 (dd, *J* = 12.0, 4.4 Hz, 2H), 4.17 (dd, *J* = 12.0, 5.8 Hz, 2H), 3.51 (t, *J* = 6.8 Hz, 2H), 2.88 (t, *J* = 6.6 Hz, 2H), 2.75 (t, *J* = 6.6 Hz, 2H), 2.52 (s, 3H), 2.29 (t, *J* = 7.6 Hz, 4H), 2.22 (s, 3H), 2.05 (t, *J* = 7.4 Hz, 2H), 1.65 – 1.57 (m, 4H), 1.50 (s, 6H), 1.37 – 1.20 (m, 48H), 0.88 (t, *J* = 6.9 Hz, 6H); ^13^C NMR (101 MHz, CDCl_3_) δ 173.5 (2C; C), 171.71 (C), 171.70 (C), 149.8 (C), 138.5 (C), 136.3 (C), 133.9 (C), 132.6 (CH), 123.2 (CH), 69.8 (CH), 62.0 (2C; CH_2_), 60.5 (CH_2_), 45.9 (CH_2_), 39.2 (C), 34.1 (2C; CH_2_), 32.1 (2C; CH_3_), 32.0 (2C; CH_2_), 29.84 (CH_2_), 29.80 (6C; CH_2_), 29.77 (4C; CH_2_), 29.72 (2C; CH_2_), 29.6 (2C; CH_2_), 29.5 (2C; CH_2_), 29.4 (2C; CH_2_), 29.2 (2C; CH_2_), 28.9 (CH_2_), 25.5 (CH_3_), 24.9 (2C; CH_2_), 22.8 (2C; CH_2_), 20.3 (CH_3_), 14.2 (2C; CH_3_); ESI-HRMS: calcd. for C_52_H_90_NaO_9_ [M + Na^+^] 881.6477; found 881.6489.

*Synthesis of* **12:** Pyridinium chlorochromate (PCC, 30.1 mg, 0.139 mmol) was added to a suspension of alcohol **11** (59.9 mg, 0.0697 mmol) and Celite (30 mg) in CH_2_Cl_2_ (3 mL) at 0 °C and the mixture stirred at rt for two hours. The reaction was filtered through a short pad of silica gel, eluting with 50% ethyl acetate/hexanes (50 mL), and the filtrate concentrated under reduced pressure to give crude aldehyde **12** (59.8 mg, quant.) as a yellow oil that was used without purification; ^1^H NMR (400 MHz, CDCl_3_) δ 9.54 (t, *J* = 2.6 Hz, 1H), 6.84 (d, *J* = 2.0 Hz, 1H), 6.60 (d, *J* = 1.4 Hz, 1H), 5.28 (m, 1H), 4.30 (dd, *J* = 12.0, 4.3 Hz, 2H), 4.16 (dd, *J* = 12.0, 5.8 Hz, 2H), 2.86 (t, *J* = 6.7 Hz, 2H), 2.83 (d, *J* = 2.6 Hz, 2H), 2.75 (t, *J* = 6.3 Hz, 2H), 2.53 (s, 3H), 2.30 (t, *J* = 7.6 Hz, 4H), 2.23 (s, 3H), 1.64 – 1.58 (m, 4H), 1.56 (s, 3H), 1.55 (s, 3H), 1.32 – 1.22 (m, 48H), 0.88 (t, *J* = 6.9 Hz, 6H).

*Synthesis of* **13:** Potassium permanganate (12.2 mg, 76.7 μmol) in 1:1 acetone/water (1.6 mL total) was added to aldehyde **12** (59.8 mg, 69.7 μmol) in acetone (1.6 mL) and the mixture stirred at rt for 17 hours. The reaction was diluted with water (10 mL), acidified to pH 2 using 1 M HCl, and the aqueous layer extracted with CH_2_Cl_2_ (3 × 15 mL). The combined organic extracts were washed with brine (40 mL), dried (MgSO_4_) and concentrated under reduced pressure to give the crude product. Purification by silica gel chromatography (10% to 25% ethyl acetate/hexanes) gave acid **13** (30.4 mg, 50%) as a colourless solid; ^1^H NMR (400 MHz, CDCl_3_) δ 6.81 (d, *J* = 1.6 Hz, 1H), 6.58 (d, *J* = 1.4 Hz, 1H), 5.28 (m, 1H), 4.30 (dd, *J* = 11.9, 4.4 Hz, 2H), 4.16 (dd, *J* = 12.0, 5.8 Hz, 2H), 2.88 (t, *J* = 6.6 Hz, 2H), 2.84 (s, 2H), 2.75 (t, *J* = 6.6 Hz, 2H), 2.53 (s, 3H), 2.29 (t, *J* = 7.6 Hz, 4H), 2.22 (s, 3H), 1.64 – 1.58 (m, *J* = 9.3 Hz, 4H), 1.57 (s, 6H), 1.34 – 1.20 (m, 48H), 0.88 (t, *J* = 6.8 Hz, 6H); ^13^C NMR (101 MHz, CDCl_3_) δ 176.1 (C), 173.6 (2C; C), 171.6 (C), 171.4 (C), 149.5 (C), 138.2 (C), 136.5 (C), 133.4 (C), 132.7 (CH), 123.0 (CH), 69.8 (CH), 62.0 (2C; CH_2_), 47.6 (CH_2_), 38.8 (C), 34.1 (2C; CH_2_), 32.1 (2C; CH_2_), 31.5 (2C; CH_3_), 29.9 (CH_2_), 29.84 (6C; CH_2_), 29.80 (4C; CH_2_), 29.76 (2C; CH_2_), 29.6 (2C; CH_2_), 29.5 (2C; CH_2_), 29.4 (2C; CH_2_), 29.2 (2C; CH_2_), 29.0 (CH_2_), 25.4 (CH_3_), 25.0 (2C; CH_2_), 22.8 (2C; CH_2_), 20.4 (CH_3_), 14.3 (2C; CH_3_); ESI-HRMS: calcd. for C_52_H_88_NaO_10_ [M + Na^+^] 895.6270; found 895.6266.

*Synthesis of* **14:** 4-(Dimethylamino)pyridine (DMAP, 2.4 mg, 19.2 μmol) and EDC•HCl (9.2 mg, 48.1 μmol) were added to a solution of acid **13** (18.5 mg, 21.2 μmol) and buprenorphine (**3**) (9.0 mg, 19.2 μmol) in CH_2_Cl_2_ (1 mL) and the mixture stirred at rt for three hours. The reaction was then concentrated under reduced pressure to give the crude product. Purification by silica gel chromatography (7.5% ethyl acetate/hexanes with 0.5% Et_3_N) gave BUP prodrug **14** (22.5 mg, 88%) as a colourless oil; ^1^H NMR (401 MHz, CDCl_3_) δ 6.80 (d, *J* = 1.9 Hz, 1H), 6.61 – 6.57 (m, 2H), 6.53 (d, *J* = 8.1 Hz, 1H), 5.90 (s, 1H), 5.28 (m, 1H), 4.39 (s, 1H), 4.30 (dd, *J* = 11.9, 4.4 Hz, 2H), 4.16 (dd, *J* = 11.9, 5.8 Hz, 2H), 3.37 (s, 3H), 3.06 (ABq, 2H), 3.01 – 2.81 (m, 5H), 2.76 (t, *J* = 6.7 Hz, 2H), 2.60 (dd, *J* = 11.6, 4.8 Hz, 1H), 2.55 (s, 3H), 2.29 (t, *J* = 7.6 Hz, 4H), 2.37 – 2.18 (m, 4H), 2.21 (s, 3H), 2.10 (t, *J* = 9.8 Hz, 1H), 1.95 (td, *J* = 12.6, 5.4 Hz, 1H), 1.89 – 1.74 (m, 2H), 1.71 – 1.51 (m, 11H), 1.33 (s, 3H), 1.45 – 1.14 (m, 49H), 1.02 (s, 9H), 0.88 (t, *J* = 6.9 Hz, 6H), 0.83 – 0.61 (m, 2H), 0.54 – 0.42 (m, 2H). 0.14 – 0.07 (m, 2H); ^13^C NMR (101 MHz, CDCl_3_) δ 173.5 (2C; C), 171.5 (C), 171.3 (C), 169.5 (C), 150.0 (C), 149.5 (C), 138.1 (C), 136.5 (C), 134.4 (C), 133.7 (C), 133.4 (C), 132.7 (CH), 131.3 (C), 123.1 (CH), 122.3 (CH), 119.3 (CH), 98.1 (CH), 80.8 (C), 79.4 (C), 69.8 (CH), 62.0 (2C; CH_2_), 59.7 (CH_2_), 58.3 (CH), 52.7 (CH_3_), 47.5 (CH_2_), 46.3 (C), 44.3 (CH), 43.7 (CH_2_), 40.5 (C), 39.0 (C), 36.1 (C), 35.5 (CH_2_), 34.1 (2C; CH_2_), 33.5 (CH_2_), 32.1 (2C; CH_2_), 31.4 (CH_3_), 31.3 (CH_3_), 29.9 (2C; CH_2_), 29.84 (6C; CH_2_), 29.81 (4C; CH_2_), 29.77 (2C; CH_2_), 29.3 (2C; CH_2_), 29.5 (2C; CH_2_), 29.4 (2C; CH_2_), 29.3 (2C; CH_2_), 29.0 (CH_2_), 26.6 (CH_3_), 25.5 (CH_3_), 25.0 (2C; CH_2_), 23.4 (CH_2_), 22.8 (2C; CH_2_), 20.4 (CH_3_), 20.1 (CH_3_), 17.7 (CH_2_), 14.3 (2C; CH_3_), 9.6 (CH), 4.3 (CH_2_), 3.4 (CH_2_).

**1.6 Synthesis of BUP-TML-C5bMe-TG (16)**

4-(Dimethylamino)pyridine (DMAP, 4.1 mg, 33.1 μmol) and EDC•HCl (15.9 mg, 82.9 μmol) were added to a solution of acid **15^2^** (32.9 mg, 36.5 μmol) and buprenorphine (**3**) (15.5 mg, 33.1 μmol) in CH_2_Cl_2_ (1.5 mL) and the mixture stirred at rt for 16 hours. The reaction was then concentrated under reduced pressure to give the crude product. Purification by silica gel chromatography (5% to 10% ethyl acetate/hexanes with 0.5% Et_3_N) gave BUP prodrug **16** (37.8 mg, 84%) as a colourless oil; ^1^H NMR (401 MHz, CDCl_3_) δ 6.79 (d, *J* = 1.8 Hz, 1H), 6.59 (d, *J* = 8.1 Hz, 1H), 6.57 (d, *J* = 1.4 Hz, 1H), 6.53 (d, *J* = 8.1 Hz, 1H), 5.90 (s, 1H), 5.28 (m, 1H), 4.39 (d, *J* = 1.4 Hz, 1H), 4.30 (dt, *J* = 11.9, 3.7 Hz, 2H), 4.14 (dd, *J* = 11.9, 6.0 Hz, 2H), 3.37 (s, 3H), 3.10 – 2.95 (m, 4H), 2.87 (m, 1H), 2.70 (dd, *J* = 14.8, 5.0 Hz, 1H), 2.64 – 2.49 (m, 4H), 2.55 (s, 3H), 2.40 – 2.18 (m, 9H), 2.22 (s, 3H), 2.10 (t, *J* = 9.8 Hz, 1H), 1.97 (m, 1H), 1.88 – 1.73 (m, 2H), 1.71 – 1.53 (m, 11H), 1.35 (s, 3H), 1.44 – 1.19 (m, 49H), 1.13 (d, *J* = 6.3 Hz, 3H), 1.05 (m, 1H), 1.02 (s, 9H), 0.87 (t, *J* = 6.8 Hz, 6H), 0.79 (m, 1H), 0.66 (m, 1H), 0.53 – 0.42 (m, 2H), 0.15 – 0.07 (m, 2H); ^13^C NMR (101 MHz, CDCl_3_) δ 173.4 (2C; C), 171.4 (C), 171.3 (C), 169.4 (C), 150.0 (C), 149.5 (C), 138.2 (C), 136.4 (C), 134.4 (C), 133.7 (C), 133.5 (C), 132.6 (CH), 131.3 (C), 123.1 (CH), 122.3 (CH), 119.3 (CH), 98.1 (CH), 80.8 (C), 79.4 (C), 69.3 (CH), 62.2 (2C; CH_2_), 59.6 (CH_2_), 58.3 (CH), 52.7 (CH_3_), 47.3 (CH_2_), 46.3 (C), 44.3 (CH), 43.7 (CH_2_), 41.3 (CH_2_), 40.7 (CH_2_), 40.5 (C), 39.0 (C), 36.1 (C), 35.5 (CH_2_), 34.1 (2C; CH_2_), 33.5 (CH_2_), 32.1 (2C; CH_2_), 31.4 (CH_3_), 31.33/31.31 (CH_3_), 29.9 (CH_2_), 29.83 (6C; CH_2_), 29.79 (4C; CH_2_), 29.75 (2C; CH_2_), 29.6 (2C; CH_2_), 29.5 (2C; CH_2_), 29.4 (2C; CH_2_), 29.2 (2C; CH_2_), 27.3 (CH), 26.5 (3C; CH_3_), 25.5 (CH_3_), 25.0 (2C; CH_2_), 23.4 (CH_2_), 22.8 (2C; CH_2_), 20.4 (CH_3_), 20.1 (CH_3_), 19.9 (CH_3_), 17.7 (CH_2_), 14.3 (2C; CH_3_), 9.6 (CH), 4.3 (CH_2_), 3.4 (CH_2_).

**2. Sample preparation and HPLC-MS analysis**

**2.1 Preparation of plasma and lymph samples**

To determine BUP concentrations in plasma or lymph, 20 µl plasma or lymph samples were diluted 1:3 or 1:10 v/v with methanol respectively. The samples were then vortexed for 1 min, centrifuged at 4500 g for 5 min and 50 µl or 150 µl of supernatant analysed by HPLC-MS/MS. As the TG prodrugs were expected to undergo resynthesis with potentially multiple different available fatty acids in the enterocyte and therefore to generate a variety of glyceride derivatives of BUP for transport into the lymph, two hydrolytic methods were developed to quantify total BUP derivatives in the lymph. For analysis of the samples collected after administration of BUP-C4-TG, BUP-CE-C4-TG, BUP-TML-C4-TG and BUP-C5βMe-TG, hydrolysis of potential glyceride derivatives of BUP in lymph was achieved via addition of 100 µl 0.5 M NaOH in 1:1 (v/v) ethanol:water to 20 µl of lymph and heating at 60^o^ C for 90 min. Subsequently, 50 µl of 1 M HCl aqueous solution was added to each sample to stop the hydrolysis, and the sample was added to 1000 µl acetonitrile, vortexed for 1 min and centrifuged at 4500 g for 5 min. An aliquot of 50 µl of the supernatant was further diluted with 950 µl of methanol before analysis for total concentration of BUP by HPLC-MS/MS. For analysis of the samples collected after administration of BUP-TML-C5βMe-TG (where the above NaOH hydrolysis process was not efficient), hydrolysis of potential glyceride derivatives of BUP in lymph was achieved via the addition of 50 µl pancreatic lipase solution (200 IU/ml) to 20 µl of lymph and incubation at 37^o^ C for 15 min. Subsequently, the sample was diluted with 800 µl acetonitrile, vortexed for a further 1 min, centrifuged at 4500 g for 5 min and 150 µl of the supernatant was analysed for the monoglyceride form of BUP (i.e. BUP-TML-C5bMe-MG) by HPLC-MS/MS. Samples were measured relative to spiked standard curves in plasma and lymph that were processed in the same way.

**2.2 HPLC-MS/MS analysis**

A Shimadzu LC-MS 8050 system (Shimadzu Scientific Instruments, Kyoto, Japan) was used to quantify BUP and derivatives in SIF, plasma and lymph. It consisted of a CBM-20A system controller, a DGU-20A5 solvent degasser, two LC-30AD pumps, a SIL-30AC autosampler, a CTO-20A column oven (held at 40 ^o^C), and a triple quadrupole mass spectrometer with an electrospray ionization interface (ESI). The desolvation line (DL) and the heat block were maintained at 250 ^o^C and 400 ^o^C, respectively. Interface and detector voltages were 4.0 kV and 2.3 Kv, respectively. The nebulizing gas flow rate and drying gas flow rate were 3 l/min and 10 l/min, respectively.

For each injection, 5 µl of sample was injected onto a C18 Ascentis Express column (50 mm x 2.1 mm; 2.7 um, Supelco, United States). The mobile phase was a mixture of solvent A and B with a flow rate of 0.3 ml/min. Solvent A was milli Q water with 0.1% (v/v) formic acid, and solvent B was 100% (v/v) acetonitrile with 0.1% (v/v) formic acid. The total flow rate was 0.3 ml/min. The gradient started with 10% (v/v) of solvent B and linearly increased to 30% over 2 min, was maintained at 30% for 0.5 min, and subsequently increased to 90% over 1 min, then remained at 90% for 1 min, before returning to 10% over 0.5 min and equilibration for 1 min prior to the next injection. The total run time was 6 minutes. The ion transitions for BUP and the hydrolysis products of each prodrug are listed in Table 1.

Table 1. HPLC-MS/MS detection conditions for the analysis of BUP and hydrolysis products of prodrugs

|  | m/z ion peak for detection |
| --- | --- |
| BUP | 468.25 🡪 468.25, positive |
| BUP-C4-TG | BUP-C4-MG*: 642.35 🡪 468.25, positive  BUP-C4-acid**: 568.35 🡪 468.25, positive |
| BUP-CE-C4-TG | BUP-CE-C4-MG*: 742.40 🡪 468.25, positive  BUP-CE-C4-acid**: 668.35 🡪 468.25, positive |
| BUP-TML-C4-TG | BUP-TML-C4-MG*: 846.45 🡪 468.25, positive  BUP-TML-C4-acid**: 772.40 🡪 468.25, positive |
| BUP-TML-C5βMe-TG | BUP-TML-C5βMe-MG*: 874.50 🡪 468.25, positive  BUP-TML-C5βMe-acid **: 800.45 🡪 468.25, positive |
| BUP-C5βMe-TG | BUP-C5βMe-MG*: 670.40, positive  BUP-C5βMe-acid**: 596.35 🡪 468.25, positive |

* Proposed MG form metabolites of TG mimetic prodrugs produced via lipolysis of the TG prodrugs at position *sn*-1 and 3 of the glycerol backbone. Standards of these intermediate metabolites were not isolated due to instability during synthesis and upon storage. However, the presence of positive ions at specified m/z values that were consistent with the corresponding proton or sodium adducts, and the rapid increase and subsequent decrease of these signals, in parallel with generation of the corresponding BUP-linker-acid form and/or free BUP, during in vitro hydrolysis, supports the contention that these adducts were MG derivatives of the prodrugs. Relative change to MG form nominally estimated by fixing the maximal signal at 100%.

** Proposed acid terminated hydrolysis products produced via hydrolysis of the corresponding MG forms. Standards of these intermediate metabolites were not isolated. However, the presence of ions at specified m/z values that were consistent with the corresponding proton adducts, and the gradual increase of these signals, in parallel with disappearance of corresponding MG forms, during in vitro hydrolysis, supports the contention that these adducts were BUP-linker-acid forms of corresponding prodrugs.

**References**

1: P. H. Bentley, W. McCrae, *J. Org. Chem.* **1970**, *35*, 2082-2083

2: L. Hu, T. Quach, S. Han, S. F. Lim, P. Yadav, D. Senyschyn, N. L. Trevaskis, J. S. Simpson, C. J. H. Porter, *Angew. Chem. Int. Ed.* **2016**, *55*, 13700-13705.

3: K. L. Amsberry, A. E. Gerstenberger, R. T. Borchardt, *Pharm. Res.* **1991**, *8*, 455-461.
